# Calcitonin Gene‐Related Peptide (CGRP)‐Expressing Neurons in the External Lateral Parabrachial Area Regulate Pain‐Induced Sleep Disturbances

**DOI:** 10.1002/advs.202500325

**Published:** 2025-06-29

**Authors:** Nicole Lynch, Roberto De Luca, Richard L Spinieli, Enrico Rillosi, Renner C Thomas, Samuel Sailesh, Nishta Gangeddula, Janayna D Lima, Sathyajit S Bandaru, Elda Arrigoni, Agustin Melo‐Carrillo, Rami Burstein, Stephen Thankachan, Satvinder Kaur

**Affiliations:** ^1^ Department of Neurology Division of Sleep Medicine and Program in Neuroscience Beth Israel Deaconess Medical Center and Harvard Medical School Boston MA 02215 USA; ^2^ Boston VA Research Institute Veterans Affairs Boston Healthcare System & Department of Psychiatry Harvard Medical School West Roxbury MA 02132 USA; ^3^ Department of Anesthesia Critical Care and Pain Medicine Beth Israel Deaconess Medical Center and Harvard Medical School Boston MA 02215 USA

**Keywords:** acute pain, neural‐circuits, optogenetics, parabrachial nucleus, sleep‐loss

## Abstract

Given that sleep and pain are bidirectionally related, we investigated the neural circuits underlying pain‐induced sleep disturbances using two acute pain models. Activation of nociceptors in acute inflammatory pain (AIP) significantly reduced sleep by 45–50% in the first 6 h, with reduced sleep spindle density for 1–3 h post‐AIP. Additionally, an “optogenetic pain (Opto‐Pain)” model is implemented to produce acute peripheral pain‐induced awakenings that reduced sleep comparable to AIP. Both pain models are used to test the role of wake‐promoting neurons in the parabrachial nucleus that express Calcitonin Gene‐Related Peptide (PBel^CGRP^) in relaying nociceptive stimulus from the dorsal horn as part of the spine‐ponto‐amygdaloid tract. Blocking PBel^CGRP^ neurons with genetic ablation or optogenetic inhibition attenuated sleep loss. To dissect the PBel^CGRP^ pathways, the terminals are then optogenetically silenced post‐AIP and found the reversal of sleep disturbances in the following descending order of effectiveness: substantia innominata of the basal forebrain (SI‐BF) > central nucleus of the amygdala (CeA) > bed nucleus of the stria terminalis (BNST) > the lateral hypothalamus (LH). In SI‐BF and CeA, a similar reversal of AIP‐induced sleep loss occurred with pharmacological blocking of either CGRP or NMDA receptors. The results are relevant to emerging pain therapies aiming to attenuate sleep disturbances.

## Graphical abstract

Lynch et al., identified novel targets on the wake‐promoting pathways that can effectively treat pain‐induced sleep disturbances. The authors demonstrated that Calcitonin gene‐related peptide‐expressing neurons (CGRP) in the external lateral parabrachial nucleus (PBel^CGRP^) serve as the primary relay node for pain stimuli that cause sleep disruption, via their projections to forebrain wake areas.

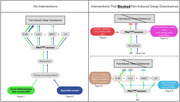

## 1 Introduction

Estimates suggest that more than 1 in 5 adults in the United States experience chronic pain, and over 70% of these individuals report ongoing sleep disturbances.^[^ ^1^, ^2^ ^]^ There is a strong bidirectional relationship between sleep and pain: chronic pain not only reduces sleep quality, but poor sleep can also lead to increased pain and predict higher pain levels.^[^ ^3^ ^]^ Frequent awakenings are the most common complaint among those with chronic pain,^[^ ^4^ ^]^ as supported by a recent study using a chronic pain model that showed increased sleep fragmentation without a reduction in total sleep time,^[^ ^5^ ^]^ suggesting that awakenings are a key driver of reported sleep disturbances and associated health consequences. One of which is heightened pain sensitivity, which can be improved with wake‐promoting agents like caffeine, or resolved with adequate sleep recovery, even despite residual sleep debt following deprivation.^[^ ^6^, ^7^ ^]^ To further examine the interactions between pain and sleep, we investigated the brain circuits relaying pain signals that disrupt normal sleep through targeted manipulations of a specific neuronal population and its corresponding projections using two different acute pain models.

The parabrachial nucleus (PB), a wake‐related node in the dorsal pons of the brainstem, is involved in integrating and relaying various sensory information to forebrain regions involved in regulating arousal and sleep‐homeostasis.^[^ ^8^, ^9^, ^10^, ^11^, ^12^, ^13^, ^14^ ^]^ PB neurons are functionally and phenotypically heterogeneous, with distinct projection patterns that are spatially separated.^[^ ^15^, ^16^, ^17^, ^18^, ^19^, ^20^ ^]^ In our investigations, we found that neurons expressing the transcription factor Fork‐head box protein (FoxP2) located in the centro‐lateral part of PB and the Kölliker‐Fuse regulate respiration and respiratory effort during apneas.^[^ ^21^ ^]^ The PB has likewise been shown to mediate arousal in response to numerous aversive sensory stimuli^[^ ^22^, ^23^, ^24^ ^]^ including in our prior work where we found that a selective cluster of glutamatergic neurons located in the external lateral PB that express the calcitonin gene‐related peptide (PBel^CGRP^) are important for cortical arousal in response to hypercapni.^[^ ^18^ ^]^ A main relay of nociception, the PB also receives pain signals directly from the dorsal horn as part of the spine‐ponto‐amygdaloid tract.^[^ ^25^, ^26^, ^27^, ^28^ ^]^ Along those lines, studies have shown that the activation of glutamatergic neurons in the PB promotes hyperalgesia and neuropathic pain in mice with a chronic peroneal nerve injury, while their inactivation provides analgesia.^[^ ^17^ ^]^ Subsequent investigations have revealed that PBel^CGRP^ neurons are not only activated by acute painful stimuli,^[^ ^22^, ^29^ ^]^ but their activity remains amplified for 5 weeks post‐exposure to neuropathic or inflammatory pain, in correlation with increased pain metrics.^[^ ^30^ ^]^

Collectively, these findings led us to hypothesize that PBel^CGRP^ neurons function as a relay node, transmitting pain stimuli to mediate cortical arousals via their forebrain projections. To investigate this, we used two acute pain models, AIP and Opto‐Pain, and examined how genetic ablation or optogenetic inhibition of PBel^CGRP^ neurons affected pain‐induced sleep disturbances. While previous studies have focused mostly on the Transient Receptor Potential Vanilloid 1 (TRPV1) receptor to stimulate selective peripheral nociceptors to cause pain behaviors,^[^ ^31^, ^32^, ^33^ ^]^ we have implemented a novel approach to wirelessly and optogenetically stimulate the CGRP‐nociceptive terminals in the foot pad to produce pain behavior (Opto‐Pain). To target CGRP terminals with optogenetics, we used CGRP‐ChR2 mice that expressed ChR2/tdTomato in all CGRP neurons and terminals, which can be activated with stimulation of blue laser light. This was done in combination with the recording of an electroencephalogram (EEG) and electromyography (EMG), which allowed us to assess sleep‐wake states and study pain‐induced awakenings.

Exploring the requisite arousal pathways, we considered prior research from our lab showing that PBel^CGRP^ neurons regulate hypercapnia‐induced arousal through their ascending projections to several forebrain areas, mainly the substantia innominata of the basal forebrain (SI‐BF), central nucleus of the amygdala (CeA), bed nucleus of the stria terminalis (BNST), and lateral hypothalamus (LH).^[^ ^18^ ^]^ Some of these sites have been previously implicated in pain transmission; however, their roles in modulating pain‐related sleep loss have not been thoroughly studied.^[^ ^16^, ^25^, ^34^ ^]^ To investigate this, we optogenetically inhibited PBel^CGRP^ terminals at each of the four sites to identify which projections are associated with pain‐induced effects on sleep. Given that PBel^CGRP^ neurons co‐release both CGRP and glutamate, and that CGRP may potentiate the effects of glutamate through NMDA receptors,^[^ ^11^, ^18^, ^22^, ^35^, ^36^ ^]^ we also administered either CGRP or glutamate receptor antagonists for local pharmacological blocking at the two terminal sites (SI‐BF and CeA) where optogenetic inhibition most effectively reversed AIP‐induced sleep loss. Our findings provide critical insight into a specific neuronal population in the brainstem that drives pain‐induced arousals, revealing key mechanisms and sites that underlie this complex neural circuitry.

## 2 Results

### 2.1 Acute Pain Model

We used two models of acute pain stimulus to study the role of the PBel^CGRP^ as relay neurons for pain‐induced arousal. Both models resulted in significant and comparable sleep loss and fragmentation.

#### 2.1.1 Acute Inflammatory Pain (AIP) Model

**Figure 1:**
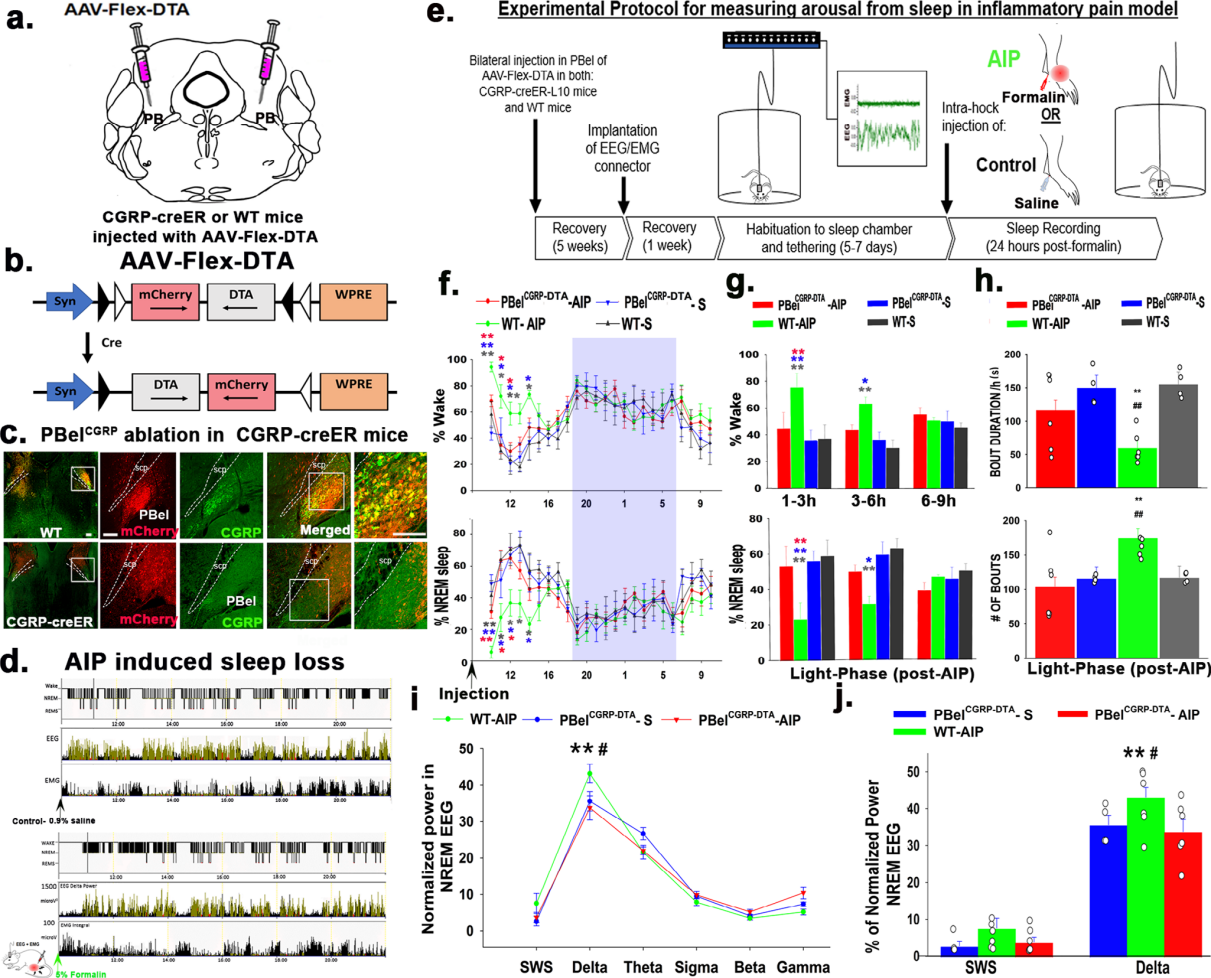
Genetic ablation of PBel^CGRP^ neurons and the resulting recovery of sleep‐loss induced by the Acute Inflammatory Pain (AIP) model: a,b) Representations depicting that CGRP‐creER and WT mice received bilateral PBel injections of the AAV‐Flex‐DTA viral vector to selectively ablate cre‐expressing CGRP neurons. c) DTA expression causes nearly complete deletion of all PBel^CGRP^ neurons (**green**) in CGRP‐creER mice (**bottom panel**), while WT mice, which lack cre recombinase, show intact CGRP neurons (**top panel**). Expression of non‐cre‐dependent mCherry (**red**) in both CGRP‐creER and WT mice confirms AAV‐Flex‐DTA expression throughout the PBel. d) Representative images showing EEG delta power, integral EMG, and hypnograms of NonREM sleep (**green**), REM sleep (**red)**, and Wake (**black**) from both Saline/Control (**top panel**) and AIP/Formalin (**bottom panel**) conditions are shown for 24 h post‐injection. AIP‐induced sleep loss (**bottom panel**) shows almost no sleep in the first hour and sleep fragmentation in the first 3 h, compared to the control above. e) The experimental protocol and timeline for the genetic ablation and AIP study are shown here. f) Average (mean ± SEM) percentage of time spent in wake and sleep, including Non‐REM (NREM) and REM, in the following treatment groups are shown across 24 h post‐injection: deletion of PBel^CGRP^ neurons following saline (PBel^CGRP^‐S) or AIP (PBel^CGRP^‐AIP), and intact PBel^CGRP^ neurons following saline (WT‐S) or AIP (WT‐AIP). Sample size = 5 and 6, for WT and PBel^CGRP‐DTA^ AIP groups, respectively, while for the S group, *n* = 4. Exact *p* values for each significance symbol are described in the results section. g) The averages (± SEM) of wake and sleep percentages across the groups in 3 h bins post‐injection during the light phase are shown here in bar graphs. Sample size = 5 and 6, for WT and PBel^CGRP‐DTA^ AIP groups, respectively, while for the S group, *n* = 4. Exact *p* values for each significance symbol are described in the results section. h) The average sleep bout duration per hour (mean ± SEM) and average number of sleep bouts (mean ± SEM) in the first nine hours post‐injection during the light phase are shown here (data points representing each mouse are included). i) A comparison of the normalized EEG power (mean ± SEM) during NREM across different frequency bins (SWS 0.5–1.5 Hz, Delta 0.5–4 Hz, Theta 5–8 Hz, Sigma 9–15 Hz, Beta 16–29 Hz and Gamma 30–60 Hz) for the groups is represented by a line graph. j) A bar graph compares the SWS and Delta NREM EEG power percentages (mean ± SEM) in the groups (data points representing each mouse are included). Groups were compared using a one‐way (treatment) or two‐way (treatment X time) ANOVA, followed by the Holm‐Sidak method for multiple comparisons, where **p < 0.001; *p < 0.01. The colors of the asterisks in (f,g) represent the group with which WT‐AIP is being compared, while in (h,j), *‐represents a comparison to PBel^CGRP‐DTA^‐ AIP, and #‐ represents a comparison to WT‐S. Exact *p* values for each significance symbol are described in the results section. Scale in (c)‐ 100 µm. *Abbreviations*: PB‐ parabrachial subnucleus; PBcl‐ central lateral PB subnucleus; PBel‐ external lateral PB subnucleus; scp‐ superior cerebellar peduncle.

In this model, animals received a single subcutaneous injection of either 5% formalin or 0.9% buffered sterile saline (25 µl) in one of the hind paws during the early light phase after a day of baseline sleep‐wake recordings (**Figure** **1**e).

#### 2.1.2 Opto‐Pain Model

**Figure 2:**
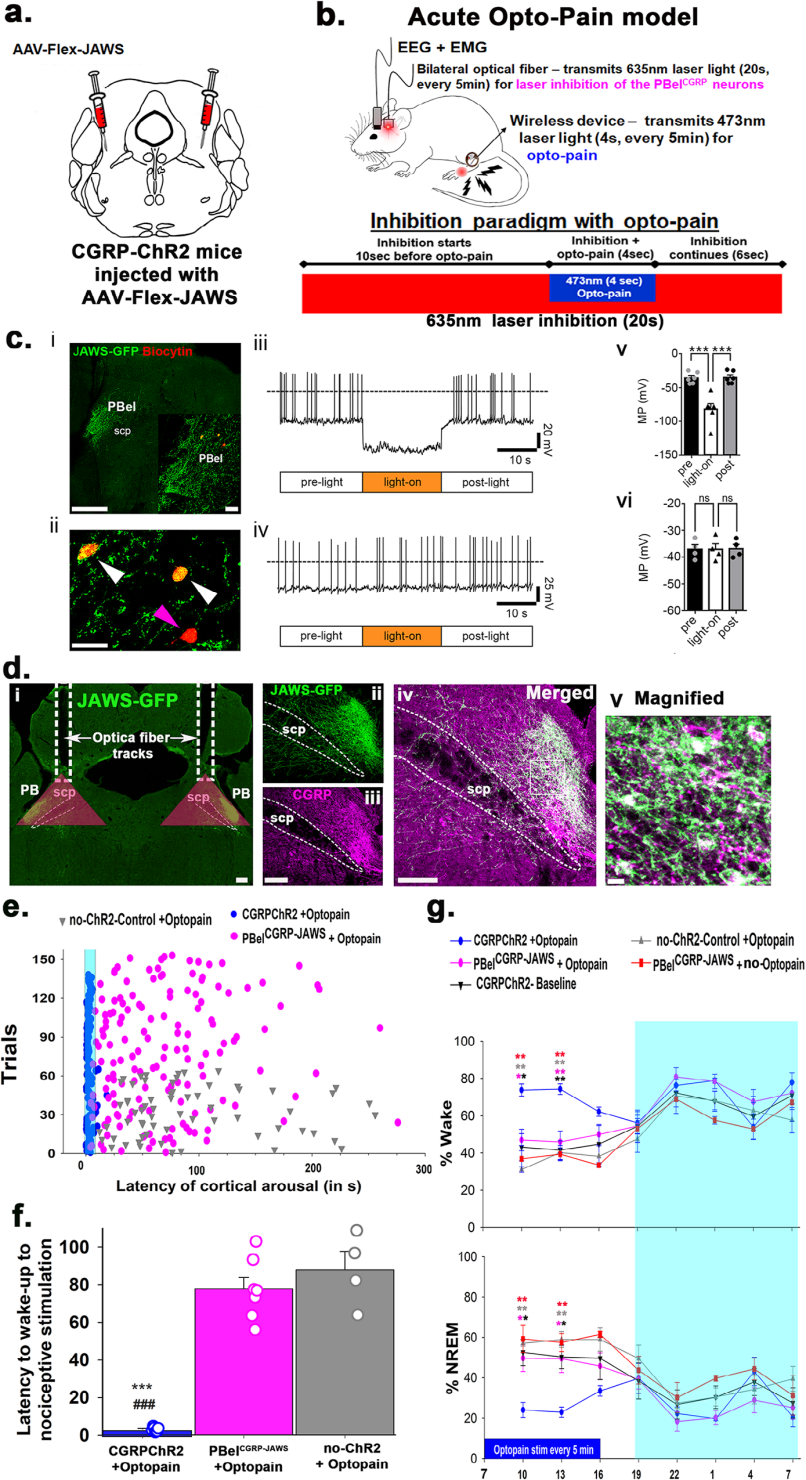
Optogenetic inhibition of PBel^CGRP‐JAWS^ neurons in the Opto‐Pain model and corresponding in vitro validation: a,b) Representations depicting that CGRP‐ChR2 mice, which express ChR2 in all the CGRP neurons and terminals (see validation in Figure S2, Supporting Information), were injected bilaterally in the PBel with AAV‐Flex‐JAWS‐EGFP (JAWS‐EGFP) and implanted with EEG/EMG sensors, bilateral optical fibers in the PBel, and a µLED wireless device in the foot pad for stimulation of the CGRP‐nociceptors for the acute opto‐pain paradigm. Details of the optogenetic inhibition of JAWS‐EGFP expressing PBel^CGRP^ (PBel^CGRP‐JAWS^) neurons and the peripheral CGRP nociceptor stimulation in the Opto‐Pain model are also included (b). c) In vitro validation of JAWS expressing PBel^CGRP^ neurons (c): (i) Examples of recorded brain slices showing JAWS‐EGFP expression in the PBel (green; scale bar: 500 µm) and *post hoc* labeling of recorded PBel neurons filled with biocytin from the recording pipette and then labeled with streptavidin‐AF‐555 conjugate (orange‐red, *insert;* scale bar: 50 µm). (ii) Image showing the same biocytin‐labeled PBel neurons at higher magnification (white arrows: two recorded PBel^CGRP‐JAWS^ ‐neurons that express JAWS‐EGFP; purple arrow: a recorded PBel neuron that does not express JAWS‐EGFP; scale bar: 50 µm). (iii) Light exposure (635 nm wavelength; 20 s duration) hyperpolarized and silenced action potential firing of PBel^CGRP‐JAWS^ neurons (*n* = 6). (v) Bar histogram graphs representing the mean membrane potential before (pre), during (light‐on), and after (post) light stimulation. One‐way ANOVA, *F=* 32.46; *p* <  0.001, *n* = 6; Bonferroni's multiple comparisons *post‐hoc* test; ***p = 0.001 pre vs light‐on; ***p = 0.001, light‐on vs post. (iv) PBel neurons that do not express JAWS (*n* = 4) do not respond to light. vi) Bar histogram graph representing no changes in the mean membrane potential (MP) before (pre), during (light‐on), and after (post) light stimulation. One‐way ANOVA, *F*= 0.21; *p* = 0.8149, *n* = 4; Bonferroni's multiple comparisons post‐hoc test; ns *p* > 0.999 pre vs light‐on; ns *p* > 0.999, light‐on vs post. d) Photomicrographs showing selective expression of JAWS‐EGFP (enhanced green fluorescent protein) in PBel^CGRP^ neurons (purple). (i) White optical fiber tracts (white dashes). These JAWS expressing CGRP cells in the PBel (**PBel^CGRP‐JAWS^**) were targeted by the implanted optical fibers (fiber tracks in (i)) to photo‐inhibit by the laser light of 635 nm (red laser), and the red shaded triangles mark the area (500^2^ µm) maximally affected by the transmitted light that includes the PBel^CGRP‐JAWS^ expressing neurons. e) The latency to cortical arousal in each trial (represented by a dot/ triangle) following peripheral CGRP nociceptors (Opto‐Pain) in CGRP‐ChR2 mice (blue; *n* = 7) was compared with mice where **PBel^CGRP‐JAWS^** neurons were photo‐inhibited by red laser light (magenta; *n* = 7), and with those that had no ChR2 expression in CGRP neurons and terminals (no‐ChR2 control; grey; *n* = 4). f) Mean (±SEM) arousal latency (in sec) for the groups shown in (e) are represented as a bar graph (data points representing each mouse are included). g) The average percent of time in wake and NREM over 24 h following the opto‐pain protocol is shown on an hourly basis for the groups in e and f. Additionally, the graphs also include two controls, CGRP‐ChR2 mice with PBel^CGRP‐JAWS^ neurons but no Opto‐Pain (red; *n* = 3), and CGRP‐ChR2 mice in a baseline condition (*n* = 4). Groups were compared using a one‐way (f) or two‐way (g) ANOVA, followed by the Holm‐Sidak method for multiple comparisons, where **, ###*p* < 0.001; *p < 0.01. The color of the asterisk in (g) represents the group being compared to CGRP‐ChR2 + Opto‐Pain, while in (f) *‐represents the comparison to **PBel^CGRP‐JAWS^+ Opto‐Pain**, and #‐ represents the comparison to **no‐ChR2 + Opto‐Pain**. Exact *p* values for each comparison are mentioned in detail in the results section. Scale in (d) (i‐iv) 100 µm, and 15 µm in magnified panel. Abbreviations: PB‐ parabrachial subnucleus; PBcl‐ central lateral PB subnucleus; PBel‐ external lateral PB subnucleus; scp‐ superior cerebellar peduncle.

In this model, we used CGRP‐ChR2 mice generated by crossing CGRP‐creER mice with Channelrhodopsin‐2 (ChR2)‐floxed mice (Ai27D mice, strain #:012567, RRID: IMSR_JAX:012567, The Jackson Laboratory). Ai27D mice express an improved ChR2/tdTomato fusion protein following exposure to cre recombinase, which can be used in optogenetic studies for rapid in vivo activation of excitable cells with blue light (473 nm). Thus, CGRP‐ChR2 mice express ChR2/tdTomato in all CGRP cells and terminals in the brain and also in peripheral tissues (sensory neurons of the dorsal root ganglia‐ DRG and sensory nerve endings in the hind foot skin; validation shown in Figure S2a–d, Supporting Information). We used these mice to selectively activate CGRP nociceptors in the hind limb, through pre‐implanted miniature devices, using blue laser light directed to the foot pad (NeuroLux, u‐iLED, 6 mm). Stimulation of the nociceptors was conducted using the NeuroLux wireless system, which creates a radiofrequency field in the cage for the duration of stimulation so that the stimulation frequency, pulse duration, and timing can be set remotely without handling or disturbing the mice. Sleep‐wake behavioral states were recorded in all mice using EEG/EMG electrodes while subjecting them to nociceptor stimulation every 5 min for 4 s, with or without simultaneous inhibition of the PBel^CGRP‐JAWS^ neurons using red laser light through pre‐implanted, bilateral, optical fibers in the PBel (635 nm). The red laser stimulus, which was used to inhibit the PBel^CGRP‐JAWS^ neurons, always preceded the nociceptor stimulation by 10 s, and lasted for 20 s, overlapping and extending past the nociceptive stimulus (**Figure** **2**b). We found that the stimulation strength of the blue laser light at 8 Hz (10 ms) activated the nociceptors, however the wakefulness was produced only in 60% of the trials with a latency of 38.5 ± 11.2 s, while 10 Hz (10 ms) for 4 s caused arousal in ≈100% of the trials with significantly shorter latency, so we continued to use the latter frequency for all the subsequent experiments.

### 2.2 Effect of Genetic Ablation of the PBel^CGRP^ Neurons on AIP‐Induced Sleep Loss

To test the role of PBel^CGRP^ neurons in awakening mice in response to pain, we conducted these experiments in CGRP‐creER mice,^[^ ^18^ ^]^ as well as their wildtype littermates that did not express the cre‐recombinase enzyme (WT). To confirm deletion, we immunolabeled the tissue sections for CGRP using an antibody raised against the CGRP peptide (Figure 1c). CGRP‐creER mice that were injected with the adeno‐associated viral vector expressing the diphtheria toxin subunit A (AAV‐Flex‐DTA; 150 nl) in a cre‐dependent manner had bilateral ablations of the PBel^CGRP^ neurons. While similar injections in the WT control mice did not kill the CGRP neurons in PBel, they expressed mCherry (Figure 1a–c). The mCherry fluorescent tag expressed with an injection of the AAV‐Flex‐DTA vector depicts the surviving, non‐cre‐expressing neurons in the PBel of both CGRP‐creER and WT mice, demonstrating the specificity and location of the genetic deletion. Our group has previously validated this transgenic mouse model and method for genetic deletions using AAV‐Flex‐DTA.^[^ ^18^, ^21^, ^37^ ^]^

In evaluating the surviving PBel^CGRP^ neurons in the PBel^CGRP^ genetic ablation group, compared to the WT group, we found that AAV‐Flex‐DTA induced bilateral deletions in 6 of the eight injected mice, while the remaining 2 had only unilateral deletions. Both groups were recorded for sleep‐wake after injection of saline (control) or 5% formalin in the hind limb (AIP) (Figure 1e). Sleep‐wake after saline (S) or formalin (AIP) was then compared between the groups with bilateral PBel^CGRP^ deletions (PBel^CGRP‐DTA^; *n* = 6) and those with intact CGRP neurons (WT; *n* = 5). Formalin (AIP) in the WT mice (*n* = 5; WT‐AIP) produced significantly higher wakefulness, 69.12 ± 1.2%, compared to 33.64 ± 3.5% in the saline injection PBel^CGRP‐DTA^ group (PBel^CGRP‐DTA^ –S), and 35.98 ± 0.28% in the WT saline injection group (WT‐S, F_3, 159_ = 13.7; *P* <0.001) in the first 6 h post‐injection. Correspondingly, the amount of NREM in the first 6 h post‐injection was significantly reduced in WT‐AIP compared to WT‐S by 53.17% (F_3, 159_ = 13.4; *p* < 0.001) or PBel^CGRP‐DTA^‐S by 55.12% (*p* < 0.001), before it returned to baseline in the last 3 h of the light phase (Figure 1f,g). Comparing the number of sleep bouts (period of NREM or REM sleep lasting at least 10 s) and amount of NREM during the light‐phase (9 h) post‐AIP suggests that AIP‐produced sleep fragmentation causes a significantly higher number of NREM sleep bouts (F_1, 16_ = 5.4; *p* = 0.033) and reduction in average bout duration (F_1, 16_ = 14.0; *p* = 0.002) compared to both saline injection groups (Figure 1h). In addition to sleep loss and fragmentation, we also examined the occurrence of sleep spindles (9–15 Hz; Figure S1b, Supporting Information) and found that AIP significantly reduced sleep spindles (F_2,33_ = 5.1; *p* <0.001) during the first 3 h post‐injection. The impact of AIP‐induced sleep loss and fragmentation is further illustrated in Figure 1d, which shows representative hypnograms of EEG with delta power and integral values of EMG in one mouse after injection of either saline or formalin, confirming that the effect of AIP is maximal within the first 3 h post‐injection.

The PBel^CGRP‐DTA^ group showed significantly less AIP‐induced sleep disruption than the WT group (CGRP intact), demonstrating a sleep loss recovery effect of 84.83% in the first 3 h (*p* <0.001) and 89.5% (*p* <0.001) in the following 3 h post‐injection during the light phase (Figure 1f,g). PBel^CGRP^ deletion also significantly reversed sleep fragmentation such that both the bout length and the number of bouts were not significantly different compared to the saline injection condition for both PBel^CGRP‐DTA^ (*p* = 0.56) and WT groups (*p* = 0.96) (Figure 1h). To investigate whether AIP‐induced sleep‐loss causes changes in EEG power, we compared the power frequencies during NREM sleep‐delta (0.5–4 Hz), and slow wave sleep, a subset range within delta (0.5–1.5 Hz), and found that AIP in the WT‐DTA (CGRP intact) condition caused a significant increase in the NREM EEG power only in the delta frequency range (F_2, 91_ = 2.38; *p* = 0.01; Figure 1i,j) over the first 3 h post‐injection. However, the increased power was not significant when averaged over the first 6 h post‐injection. Since there is clearly AIP‐induced sleep loss in the first hour, this suggests that in the subsequent 2 h, there is significant compensatory sleep rebound, as seen by the increase in delta power, despite the persisting decrease in total NREM and increased sleep fragmentation compared to control conditions. Lastly, despite no significant differences between the NREM EEG power within the spindle frequency range (sigma‐ 9–15 Hz; Figure 1i), the WT‐AIP (2.5 spindles min^−1^) group has significantly reduced sleep spindles (F_3,36_ = 3.7; *p* = 0.006) compared to WT‐saline (*p* < 0.001; 6 spindles min^−1^) and PBel^CGRP‐DTA^ ‐saline (*p* < 0.001) injection groups, which showed reversal effects in the PBel^CGRP‐DTA^ ‐AIP (*p* = 0.002; 5.9 spindles min^−1^) group (Figure S1b, Supporting Information).

### 2.3 Optogenetically Silencing PBel^CGRP^ Neurons During Acute Opto‐Pain Stimulus

After validating the CGRP‐ChR2 mice for the presence of ChR2 (co‐expression of mCherry) in CGRP neurons, their terminal sites in the brain and also in the skin of their footpads and in the lumbar DRG‐L4 (Figure S2, Supporting Information), we conducted experiments using a wireless optogenetics system (NeuroLux Inc., Champaign, IL) to activate nociceptors in the foot pad using blue laser light (473 nm) (opto‐pain model). In this model, we tested the necessity of the PBel^CGRP^ neurons to relay pain signals that cause arousal by acutely silencing these neurons using far‐red laser light through bilateral optical fibers acting on the PBel^CGRP^ neurons that expressed an inhibitory opsin, AAV‐Flex‐JAWS (PBel^CGRP‐JAWS^), as per the paradigm shown in Figure 2a.

#### 2.3.1 In Vitro Validation of JAWS Optogenetic Silencing

Using brain slices, we confirmed that the JAWS‐expressing PBel^CGRP^ neurons were inhibited by red (635 nm) laser light (Figure 2c‐iii‐vi). Continuous 20‐s red laser light exposure hyperpolarized JAWS‐expressing PBel^CGRP^ neurons (−46.70 ± 8.12 mV; *n* = 6) and silenced the neuronal firing (Figure 2c‐iii,v), whereas similar light stimulation was ineffective toward PBel neurons that did not express JAWS (−0.05 ± 0.45 mV; *n* = 4) (Figure 2c‐ iv and vi).

#### 2.3.2 In Vivo Optogenetic Inhibition

CGRP‐ChR2 (*n* = 10) or CGRP‐creER (no ChR2‐ control; *n* = 4) mice were injected with AAV‐Flex‐JAWS (150 nl) bilaterally in the PB. These mice were then implanted with EEG/EMG, bilateral optical fibers directed to the PB, and miniature probes (NeuroLux, u‐iLED, 6 mm) in the foot pad to stimulate nociceptors in the foot wirelessly and remotely, avoiding any handling of the mice (Figure 2a,b). The optical fiber track placement, as well as the specific expression of JAWS (co‐expressing EGFP) in the PBel^CGRP^ neurons, were validated and assessed with immunohistochemistry (Figure 2di–v). Out of the 10 CGRP‐ChR2 mice, we found that seven had more than 80% PBel neurons that were transfected with JAWS and optical fibers placed within 1–1.5 mm dorsal to the transfected PBel^CGRP‐JAWS^ neurons. Therefore, these seven cases were included for the sleep and latency analysis (Figure 2e–g). Of the remaining three, one had no transfection of JAWS, and the other two had off‐target fiber placement.

We found that stimulation of peripheral CGRP nociceptors in hind limb at a frequency of 10 Hz (10 ms) for a 4‐s duration wakes the mice in 100% of trials with significantly shorter latency of 2.98 ± 0.43 s, compared to the control mice without ChR2 (87.91 ± 9.6 s; F_7,15_ = 73.81; *p* < 0.001). However, when the nociceptor stimulation was preceded by 10 s of silencing the PBel^CGRP‐JAWS^ neurons (Figure 2b), arousals were prevented, and the latency of wakefulness was significantly increased (77.63 ± 0.61 s). Such silencing in the presence of red light has been validated in vitro (Figure 2c). The same optogenetic silencing in the three mice with off‐target fiber placement or no JAWS transfection did not prevent arousal to the nociceptive stimulus; the mice showed similar arousal latency to those that received Opto‐pain without PBel^CGRP‐JAWS^ inhibition.

Repeated nociceptive stimulation (10 Hz; 4 s every 5 min) with blue laser (ZT1‐ZT10) induced a significant increase in the percent time spent in wakefulness (62.9 ± 12.2%; F_4, 143_ = 14.2; *p* < 0.001) and a decrease in NREM (by 48.2 ± 7.3%; F_4, 143_ = 13.78; *p* <0.001) for the first 6 h during the light phase, compared to baseline and no‐ChR2 controls. The data from the no‐ChR2 controls confirm that the presence of ChR2 is important for stimulation of CGRP nerve endings (Figure S2d, Supporting Information) in the hindfoot to cause pain‐induced arousal.

Inhibiting the PBel^CGRP^ neurons using a red laser without any nociceptor stimulation (PBel^CGRP‐JAWS^ + no opto‐pain group) did not change the percent time spent in the wake or NREM, which was comparable to the baseline sleep‐wake condition, demonstrating that the PBel^CGRP^ control inhibition is not inducing sleep, but is only preventing otherwise prompted arousals. However, simultaneous inhibition of PBel^CGRP‐JAWS^ neurons using red laser exposure with nociceptor stimulation (PBel^CGRP‐JAWS^+ opto‐pain) recovered the opto‐pain induced NREM sleep loss by 95 ± 1.7% (*p* = 0.004 in 1–3 h, and *p* = 0.002 in 3–6 h post‐stimulation onset, Figure 2g) and significantly reduced the opto‐pain induced wakefulness (*p* = 0.002 in 1–3 h, and *p* < 0.001 in 3–6 h post‐stimulation onset).

### 2.4 Opto‐Inhibition of the PBel^CGRP^ Terminal Sites During AIP‐Induced Sleep Loss

**Figure 3:**
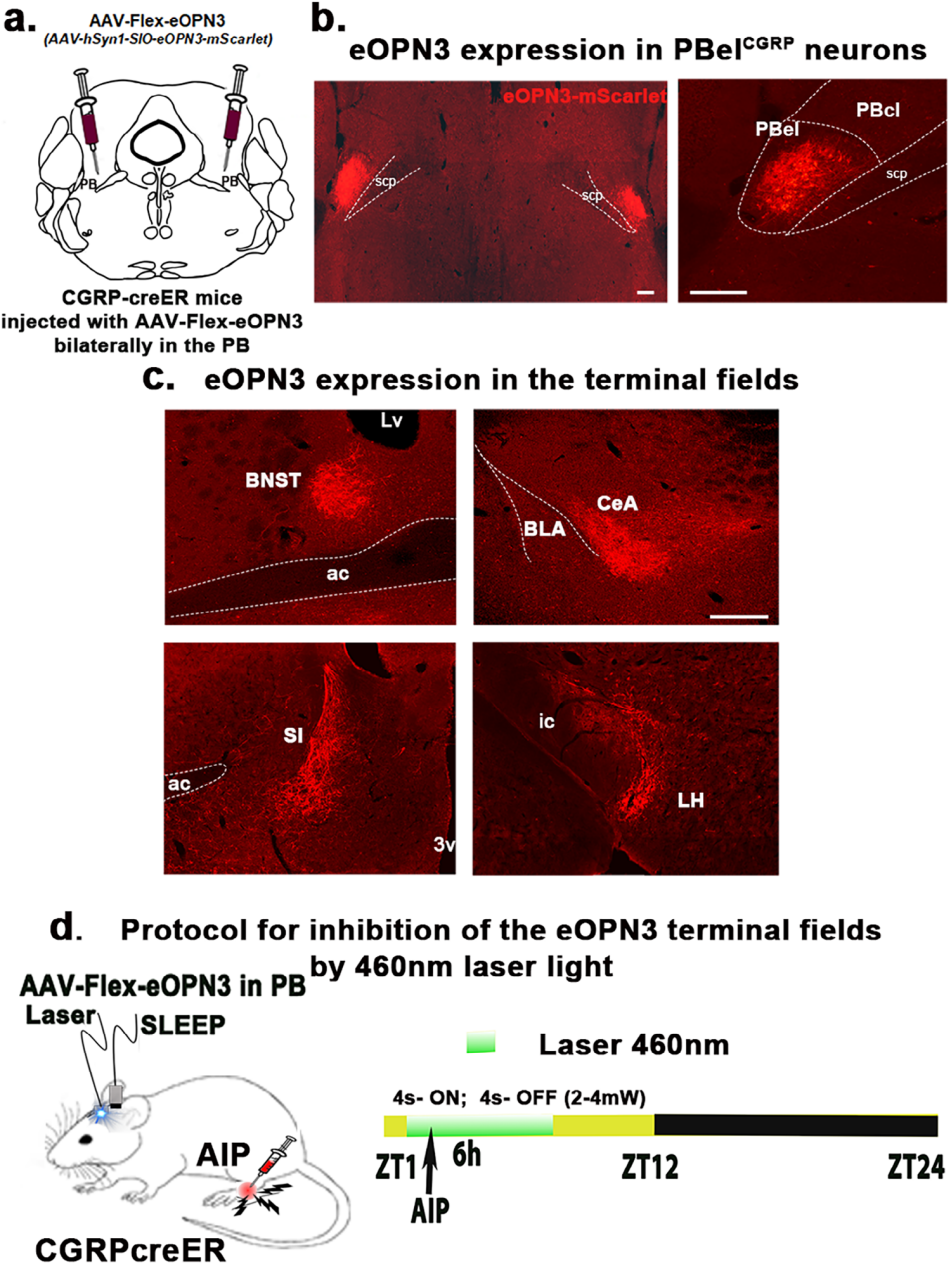
Expression of eOPN3 in the terminal fields of the PBel^CGRP^ neurons. a–c) CGRP‐creER mice were injected bilaterally in the PBel with the cre‐dependent viral vector that co‐expresses eOPN3 (AAV‐hSyn1‐SIO‐eOPN3‐mScarlet) and mScarlet. b,c) eOPN3 expression is shown in the PBel^CGRP^ neurons, and their terminals in the corresponding projection sites are immunolabeled for dsRed (scale: 100 µm). d) Protocol for optogenetic inhibition of PBel^CGRP^ neuron terminals in the AIP model, which blocks the excitatory input from the PBel^CGRP^ neurons at the targeted site. *Abbreviations*: ac, anterior commissure; SI, substantia innominata; BNST, bed nucleus of stria terminalis; ic, internal capsule; CeA, central nucleus of amygdala; LH, lateral hypothalamus; PBcl, central lateral PB subnucleus; PBel, external lateral PB subnucleus; scp, superior cerebellar peduncle.

Next, we tested which of the forebrain terminal fields of PBel^CGRP^ neurons relays nociceptive information to cause arousal by selectively opto‐inhibiting each of the four main terminal fields, SI‐BF, CeA, BNST, or LH. We used a cre‐dependent inhibitory opsin virus (AAV‐hSyn1‐SIO‐eOPN3‐mScarlet, also called AAV‐Flex‐eOPN3) that suppresses or blocks the excitatory input from the PBel^CGRP^ neurons when the terminal sites are exposed to the laser light (**Figure** **3**), causing selective silencing of the presynaptic terminal fields, as has been demonstrated previously.^[^ ^38^, ^39^, ^40^ ^]^ AAV‐Flex‐eOPN3 provides long‐lasting, reversible inhibition of synaptic transmission through direct activity on the presynaptic release apparatus and Ca^2+^ channel function and is an effective tool for long‐term inhibition, without causing tissue damage, as it only requires brief light pulses at low power.^[^ ^38^, ^39^ ^]^

**Figure 4:**
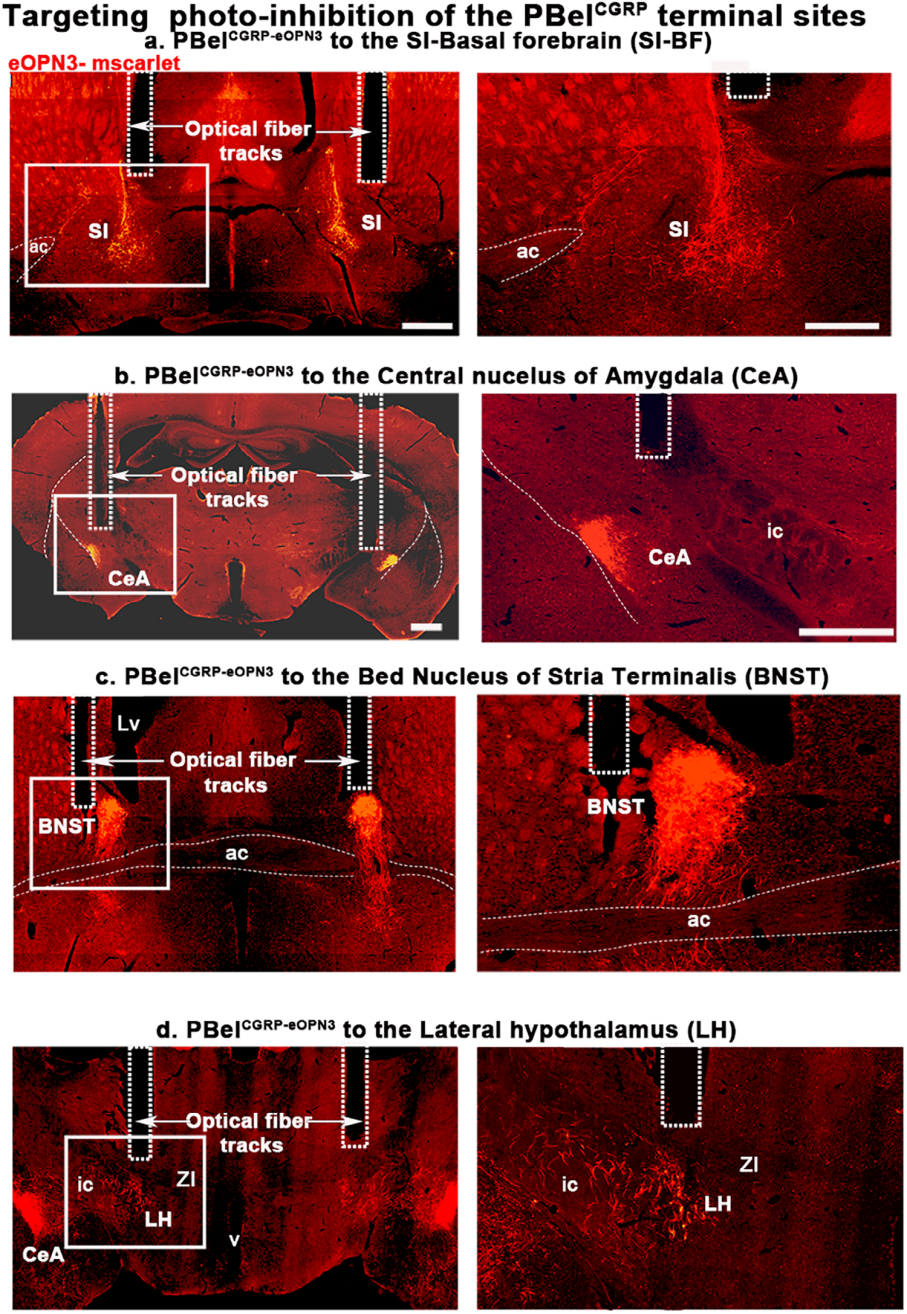
Optical fiber tracts in forebrain arousal areas for optogenetic inhibition of PBel^CGRP^ terminals: Photomicrographs showing representative brain sections of eOPN3 expression in the terminal fields of the PBel^CGRP^ neurons (PBel^CGRP‐eOPN3^) and the bilateral fiber tracts of the corresponding optical fibers (dashed rectangles) in the (a) substantia innominata (SI) of the basal forebrain (BF), (b) central nucleus of the amygdala (CeA) (b), bed nucleus of the stria terminalis (BNST) (c), and the lateral hypothalamus (d). Dashed rectangles in (a–d) marked the optical fiber tracks that illuminated the terminal sites with LED light (460 nm) at a power of 2–4 mW. Scale in a = 500 (left) and 250 (right) µm and b–d = 200 µm. *Abbreviations*: ac‐ anterior commissure; SI‐ substantia innominata; BNST‐ bed nucleus of stria terminalis; ic‐ internal capsule; CeA‐ the central nucleus of the amygdala; LH‐ lateral hypothalamus.

**Figure 5:**
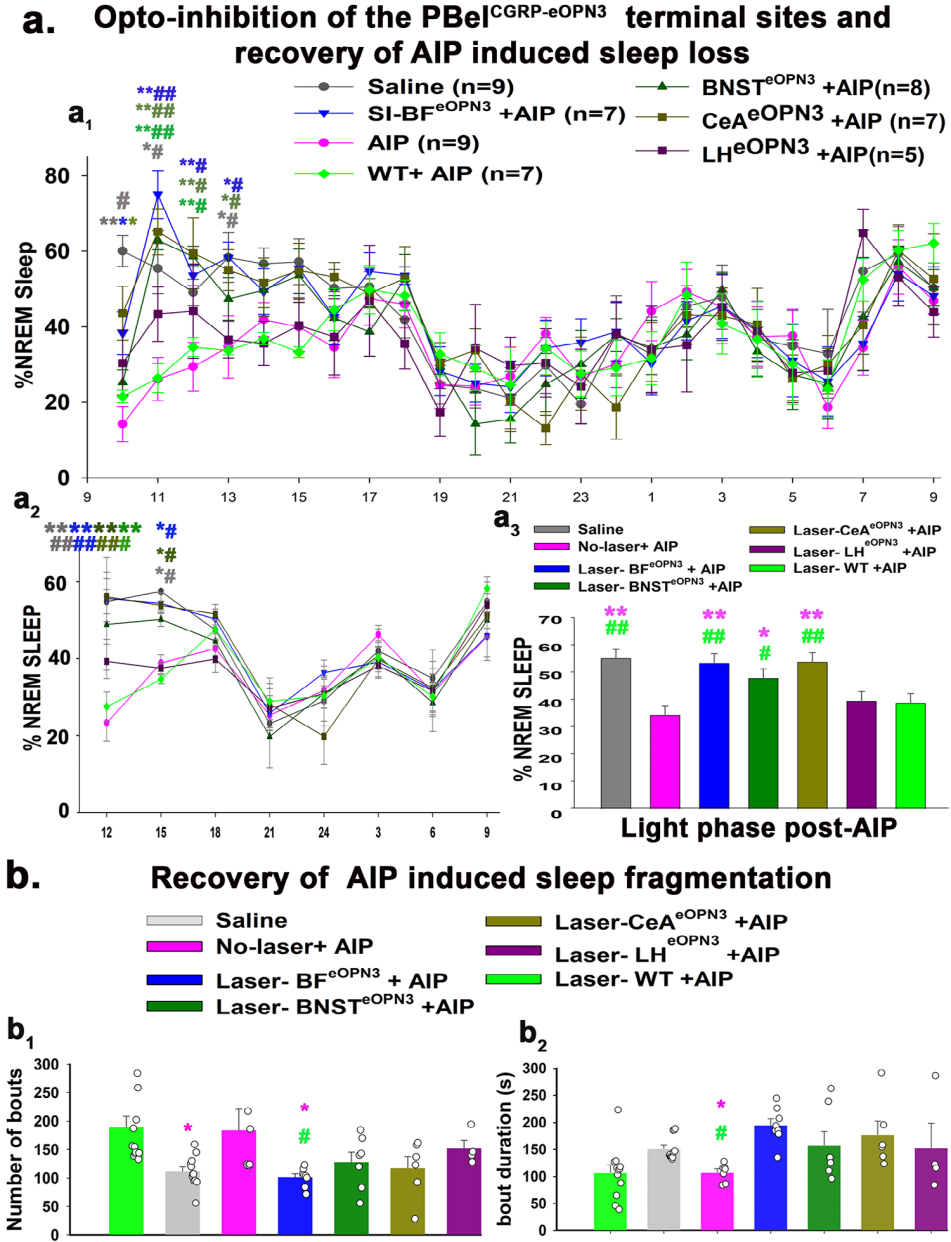
Recovery of AIP‐induced sleep loss with optogenetic inhibition of PBel^CGRP‐eOPN3^ terminals: a_1_) Percentage (mean ±SEM) of the time spent in the NREM sleep on an hourly basis over 24 h post AIP (*n* = 9) or control (saline; *n* = 9) compared across the seven conditions: Saline in CGRP‐creER, AIP in CGRP‐creER mice, AIP in WT mice, AIP with optogenetic inhibition of PBel^CGRP‐eOPN3^ terminals in the SI‐BF (BF^eOPN3;^ *n* = 7) or in the CeA (CeA^eOPN3;^ *n* = 7), or in the BNST (BNST^eOPN3;^ *n* = 8), or in the LH (LH^eOPN3^; *n* = 5). Wildtype mice (WT) that had a similar injection of the viral vector in the PB, but it did not transduce the PBel^CGRP^ neurons, were also recorded for sleep in response to AIP with laser illumination (WT+AIP; *n* = 7). a_2_) Data averaged (Mean ± SEM) over 3 h bins during the light phase. a_3_) Bar graphs show the averaged NREM (Mean ± SEM) from the same groups as in a_1_ and a_2_ over the light phase post‐AIP. Comparison of sleep showed that the order of effect of photoinhibition on sleep reversal was SI‐BF> CeA> BNST> LH. b) Bar graphs showing the recovery in AIP‐induced sleep fragmentation, with individual data points for each mouse included. The sleep fragmentation is assessed by the number of NREM bouts (mean ± SEM; b1) and average sleep bout durations (mean ± SEM; in sec) (b2) for the groups described above, during the light phase post‐injection. Groups were compared using a two‐way (a_1_, a_2_) or one‐way (a_3_‐b_2_) ANOVA, followed by the Holms‐Sidak method for multiple comparisons, where **, ##*p* < 0.001; *, #*p* < 0.01. The color of the asterisk represents the group being compared with the **AIP**, while # ‐represents a comparison to WT+AIP (a_1_‐a_2_). The color of * and # in a_3_–b_2_ represents the comparison to AIP and WT‐AIP, respectively. Exact *p* values for each comparison are mentioned in detail in the results section.

Injections of AAV‐Flex‐eOPN3 were made bilaterally in the PB of CGRP‐creER mice (*n* = 37) and WT mice (no cre expression; *n* = 7), creating PBel^CGRP‐eOPN3^ mice that express eOPN3 in a cre‐dependent manner in PBel^CGRP^ neurons and their terminal fields (**Figures** 3b,c and **4**a–d), while WT mice had no eOPN3 expression. After recovery, all mice were implanted with bilateral optical fibers targeting one of the terminal fields: the SI‐BF (*n* = 11), the CeA (*n* = 8), the BNST (*n* = 8), or the LH (*n* = 8) (Figure 4a–d). To validate eOPN3 expression in the terminal fields of the CGRP‐creER mice, we immunostained the brain sections for dsRed (mScarlet eOPN3 fluorescent tag) and verified the placement of the bilateral optical fiber tracts. Of the 37 mice PBel^CGRP‐eOPN3^ mice, eight mice had optical fibers targeting the SI‐BF, seven mice the CeA, seven mice the BNST, and five mice the LH. These mice also had more than 80% transfection of the CGRP cells in the PBel, which was confirmed with corresponding transfection in the terminal fields (Figure 4a–d). We compared each of these groups to the WT group following AIP (injection of 5% formalin in the hind limb) and saline injection, with or without the opto‐inhibition of the PBel^CGRP^ terminals as per the paradigm (Figure 3d). We also investigated which of the terminal sites contributed to PBel^CGRP^‐mediated regulation of AIP‐induced sleep loss (Figure **5**a) and sleep fragmentation (**Figure** 5b).

AIP in WT and PBel^CGRP‐eOPN3^ mice (without opto‐inhibition) induced significant sleep loss of 45% (F_6,405_ = 23.0; *p* < 0.001) in the first 6 h compared to saline injection control. AIP also induced sleep fragmentation in the first 9 h compared to saline injection, where there was a significant increase in the number of sleep bouts (F_6, 49_ = 3.9; *p* = 0.003) and a decrease in the bout duration (F_6, 49_ = 4.13; *p* = 0.002).

For opto‐inhibition of the PBel^CGRP^ terminal sites, blue laser light (460 nm) was turned on continuously for 4 s, then off for 4 s, and this pattern was then repeated for a duration of 6 h (ZT2‐ZT8) during the light phase following either AIP or saline injection. Both PBel^CGRP‐eOPN3^ and WT mice were recorded for four conditions in randomized order: 1) sleep‐wake after saline injection, 2) sleep‐wake after AIP, 3) sleep‐wake after saline injection + 6 h of opto‐inhibition, and 4) sleep‐wake after AIP + 6 h of opto‐inhibition, with one week of recovery in between each recording. Of the four terminal sites, we found that PBel^CGRP‐eOPN3^ inhibition of SI‐BF (SI‐BF^eOPN3^) and CeA (CeA^eOPN3^) had very similar recovery of AIP‐induced sleep loss (F_6, 147_ = 67.42; *p* < 0.001; SI‐BF‐ 98% and CeA‐ 97.95%), followed by PBel^CGRP‐eOPN3^ inhibition of BNST (BNST^eOPN3^; 88.3%; *p* = 0.022) in the first 1–6 h compared to AIP in both PBel^CGRP‐eOPN3‐^ no laser and WT groups (Figure 5a2). Opto‐inhibitions of the LH (LH^eOPN3^) showed a trend toward recovery of AIP‐induced sleep loss (14.8%), but it was not statistically significant (*p* = 0.85).

The inhibition of terminals at both the SI‐BF and the CeA caused significant recovery in AIP‐induced sleep loss as compared to both WT‐AIP and PBel^CGRP‐eOPN3^‐no‐laser‐AIP (no opto‐inhibition) and WT‐AIP throughout the first 4 h post‐injection (Figure 5a_1_; F_6,405_ = 5.9; *p* < 0.001). Terminal inhibition in the BNST caused significant recovery in AIP‐induced sleep loss in the second (*p* < 0.001) and third (*p* < 0.001 and *p = 0.002*, compared to AIP and WT‐AIP) hours post‐injection. Averaging sleep across 3‐h bins post‐injection (Figure 5a2) also showed that both SI‐BF and CeA had comparable AIP‐induced sleep loss recovery (F_6,112_ = 3.55; *p* = 0.003), which was significant for the first (*p* < 0.001) and second (*p* = 0.028‐ SI‐BF; *p* = 0.0035‐CeA) 3 h bins, while AIP‐induced sleep loss recovery in the BNST was only significant for the first 3‐h bin (*p* < 0.001 and *p* = 0.008). These results suggest that PBel^CGRP^→ SI‐BF + CeA is presumably the pathway that causes awakenings to pain, with a smaller contribution of PBel^CGRP^ projections to the BNST, and, finally, PBel^CGRP^ projections to the LH contributing the least amount.

### 2.5 Pharmacological Blocking at PBel^CGRP^ Terminal Sites During AIP‐Induced Sleep Loss

We used local pharmacological blocking to understand if pain‐induced arousal stimulus is relayed through the CGRP or the glutamatergic NMDA receptors, as all PBel^CGRP^ neurons are glutamatergic^[^ ^18^ ^]^ and their terminal sites express both CGRP and NMDA receptors.^[^ ^35^, ^41^, ^42^ ^]^ For this, we implanted bilateral guide cannulas (26G, Plastics One) in C57bl/6J mice targeting PBel terminal fields in the SI‐BF (*n* = 10) and CeA (*n* = 8). We chose these two sites because opto‐inhibition at these PBel^CGRP^ terminal sites was most effective in preventing pain‐induced arousals. For the pharmacological blocking, we injected CGRP receptor blocker, BIBN (BIBN4096BS; 80 µg mL^−1^; Boehringer Ingelheim Pharma KG, Biberach, Germany) and NMDA receptor blocker injection, AP‐5 (40 µg mL^−1^), which were delivered using the injector cannula that was placed in the guide and extending past the tip of the guide by 1 mm. Based on previous studies, we chose the dosages for BIBN^[^ ^43^, ^44^ ^]^ (0.016 µg mouse^−1^) and NMDA^[^ ^45^ ^]^ (0.2 µg/ 0.5 µl), which are comparatively lower than those used earlier, so as to avoid their nonspecific adverse effects on sleep. In addition, we chose a total injection volume of 200 nl on each side to restrict the spread of the drug, which was administered bilaterally at 3.3 nl s^−1^ simultaneously, using a programmable dual syringe pump (Harvard Apparatus, MA, US). The bilateral injections were completed within 1–2 min after insertion of the injector cannula, with minimum handling.

**Figure 6:**
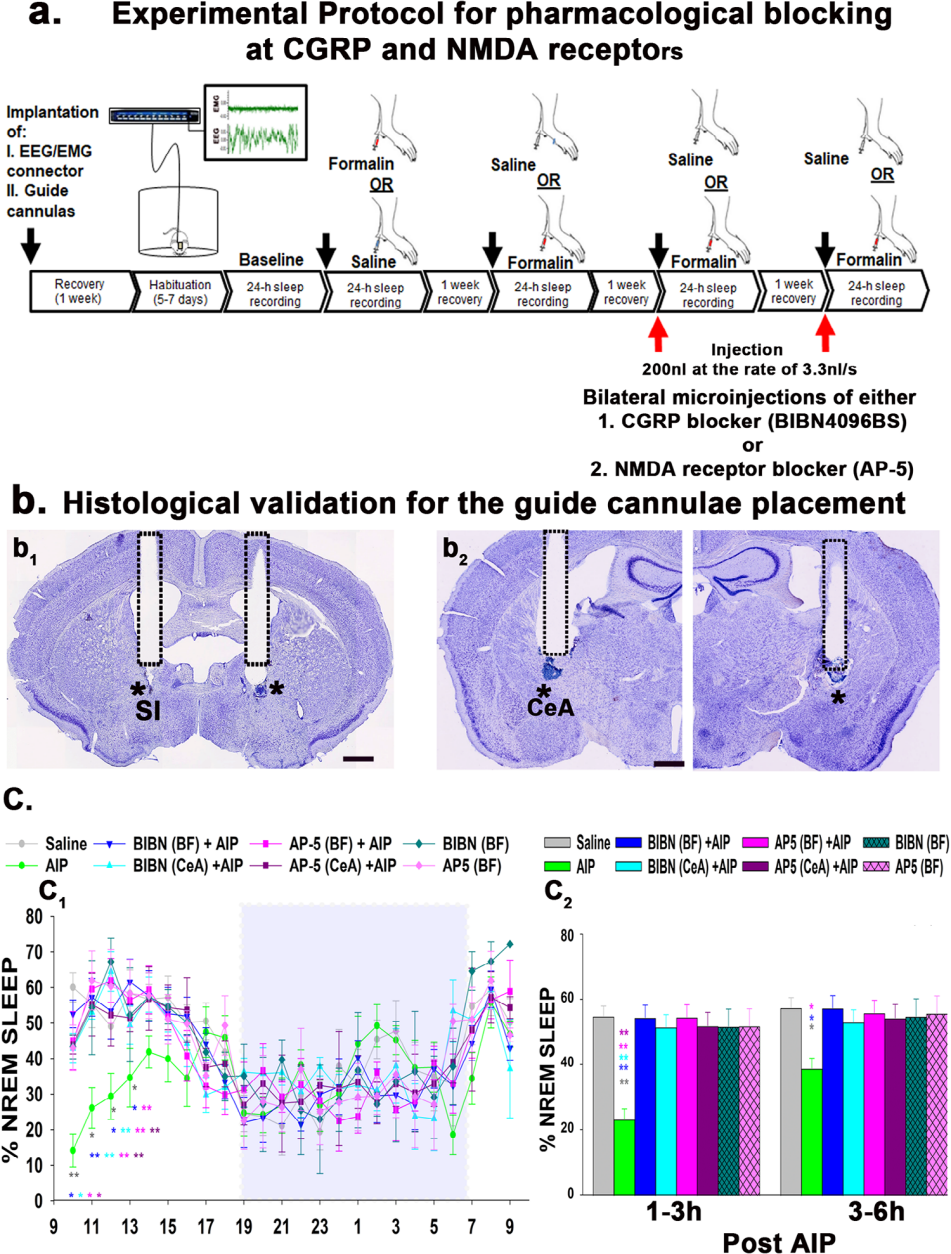
Recovery of AIP‐induced sleep loss with pharmacological blocking of SI‐BF or CeA using CGRP or NMDA receptor antagonists: a) Experimental timeline and protocol for sleep/wake recordings of WT mice during either Formalin (AIP) or Saline (control), with or without local brain injection of either CGRP (BIBN4096BS; 80 µg µl^−1^) or NMDA (AP‐5; 40 µg µl^−1^) receptor antagonist, using a programmable dual syringe pump connected to a guide cannula implanted bilaterally in either the SI‐BF or CeA. Both pharmacological blockers were administered using a 10 µl Hamilton syringe at a rate of 3.3 nl s^−1^ for a total volume of 200 nl. b) Photomicrographs of Nissl‐stained brain sections with the tracks of the guide cannulas (black dashes) used for insertion of the injector cannulas, the tips of which have been marked with additional thionine stain to show vicinity (within a radius of 1–1.5 mm; scale: 0.5 mm) to the substantia innominata (SI‐BF; b_1_) and the central nucleus of the amygdala (CeA, b_2_). c) Bar graphs show the percentage (mean ± SEM) time in NREM on an hourly basis over 24 h (c_1_), and in 3 h bins over the first 6 h (c_2_), after either AIP or Saline (control). The shaded box represents the active/ dark phase. Sample sizes for the graphs in c_1_ and c_2_ are, Saline, *n* = 9; AIP, *n* = 9; BIBN^BF^ +AIP, *n* = 6; BIBN^CeA^ +AIP, *n* = 5; AP5^BF^ +AIP, *n* = 6; AP5^CeA^ +AIP, *n* = 5; BIBN^BF^, *n* = 3; AP5^BF^, *n* = 3. Groups were statistically compared using a two‐way (treatment X time) ANOVA, followed by the Holms‐Sidak method for multiple comparisons, where **p < 0.001; *p < 0.01. The color of the asterisk represents the group being compared to the AIP group. Exact *p* values for each comparison are mentioned in detail in the results section.

Sleep‐wake was then recorded for 24 h in these mice under the six treatment conditions in randomized order: 1) sleep‐wake after saline injection, 2) sleep‐wake after AIP, 3) sleep‐wake after BIBN, 4) sleep‐wake after AP‐5, 5) sleep‐wake after AIP + BIBN, 6) sleep‐wake after AIP + AP‐5, with one week of recovery in between each recording. After all recordings were completed, all mice received bilateral injections of thionine (50 nl) using the same equipment for histological confirmation of injector cannula placement (**Figure** **6**b). Of the 18 total mice, six mice that were implanted with bilateral cannulas in the SI‐BF, and five that were implanted with bilateral cannulas in the CeA had cannula track and injector tip marks (indicated by thionine stain) within 1–1.5 mm in the vicinity of the corresponding terminal sites (as seen by eOPN3 staining in Figure 4a–d), and these were identified based on the anatomical landmarks (as shown in Figure 6b). The remaining seven cases, where the cannula tips were 3–4 mm away from the terminal sites were considered anatomical controls, where the injections of the blockers did not prevent AIP‐induced sleep loss.

AIP alone induced significant sleep loss of 57.6% (F_7, 128_ = 50.75; *p* < 0.001) in the 1–3 h period and 32.4% (*p* = 0.047) in the subsequent 3–6 h period post‐injection (Figure 6c) compared to saline injection. We then compared the percentage of time spent in sleep after local injections of AP5 or BIBN blockers with AIP treatment, with those of either saline injections or injections of blockers alone. BIBN injections (CGRP antagonist) recovered AIP‐induced sleep loss in the SI‐BF implanted mice by 97.4%, and 93.8% in the CeA implanted mice in the 3–6 h period post‐injection. We found that the NMDA receptor blocker (AP‐5) effects were comparable to the BIBN at both injection sites during the 1–3 h and 3–6 h periods post‐injection. Injections of either BIBN or AP‐5 alone (no AIP) in the SI‐BF mice did not increase NREM, such that the sleep‐wake amounts post‐injection were comparable to the saline condition, demonstrating that the receptor blockers do not specifically promote sleep (Figure 6c), but instead block the relay of pain stimulus that leads to wakefulness. Our results also suggest that PBel^CGRP^ neurons may act on both the CGRP and NMDA receptors at the SI‐BF and CeA sites, where CGRP may potentiate the effect of glutamate by acting on NMDA receptors.^[^ ^35^ ^]^

## 3 Discussion

In the present study, we tested the hypothesis that PBel^CGRP^ neurons act as a relay node for nociceptive stimuli, mediating pain‐induced wakefulness. Our findings, using two acute pain models that both reliably induced objectively quantifiable sleep loss, show a reduction in sleep spindle density and increased sleep fragmentation that lasted for 3–6 h, providing significant insights into the mechanisms of pain‐induced sleep disruption.

In pain research, formalin administration (AIP) in mice or rats is a valid and reliable model of tonic pain and nociception, and is often used to assess the analgesic potential of various compounds.^[^ ^46^, ^47^, ^48^, ^49^, ^50^, ^51^ ^]^ A subdermal injection of formalin in the hindlimb produces a typical biphasic time course of pain behaviors lasting at least an hour. The first phase of resulting pain behavior is due to direct stimulation of nociceptors in the hindlimb. The second and longer‐lasting phase is mediated by a combination of peripheral input and spinal cord sensitization that depends on the extent of peripheral inflammation. This response is unlike that with the TRPV1 receptor agonist capsaicin, which does not activate the dorsal horn and has only shorter and milder nociceptive responses.^[^ ^52^, ^53^ ^]^ Therefore, the more prolonged tonic pain induced by formalin is more consistent with acute pain from trauma and injury and is more likely to cause sleep changes seen in patients with acute peripheral injuries.^[^ ^54^, ^55^, ^56^, ^57^, ^58^ ^]^

Several studies have investigated the relationship between sleep fragmentation and increased pain sensitivity,^[^ ^6^, ^7^, ^59^, ^60^, ^61^ ^]^ while others have linked inflammation to sleep disruption.^[^ ^62^, ^63^, ^64^, ^65^, ^66^, ^67^ ^]^ Still, none have attempted to objectively investigate if acute pain in AIP models reliably produces quantifiable sleep loss and fragmentation, which can be used to detect analgesic efficacies of various compounds and identify pathways that modify this pain‐induced sleep loss. Therefore, we first characterized sleep‐wake responses in the AIP model during the early light phase by objectively recording and analyzing sleep‐wake and comparing them with control conditions. Our results suggest that AIP resulted in a robust and consistent reduction of sleep by 45–50% over the first 6 h, with the most significant reduction occurring in the first hour (by 89%), which also matches the timing of heightened pain behaviors observed in rodents.^[^ ^52^, ^53^ ^]^ This period was followed by a significant increase in NREM delta power (0.5–4.0 Hz) in the following 2–3 h post‐AIP, suggestive of a sleep‐rebound response to a near‐total sleep loss during the first hour. We also observed significantly fragmented sleep that lasted for the remainder of the light‐phase post‐AIP (9 h), which is consistent with observations in other pain models of neuropathic injury.^[^ ^5^ ^]^ Lastly, despite no change of the EEG power in the spindle frequency range (sigma‐ 9–15 Hz), we found that AIP also significantly reduced sleep spindle density for 1–3 h post‐AIP, which aligns with other studies in rodents^[^ ^68^ ^]^ and humans^[^ ^69^, ^70^ ^]^ that suggest spindle density negatively correlates with pain intensity and sensitivity.

Additionally, we employed an opto‐pain model that involved the precise and selective activation of peripheral nociceptors and primary afferents expressing CGRP in the hindlimb via laser light (10 Hz, 10 ms). This model produced brief arousals with short latency compared to the control condition, suggesting that arousals were selective to the activation of the CGRP‐expressing primary afferents. Repeated stimulations of nociceptors in the opto‐pain model simulated acute peripheral pain‐induced awakenings, which, during the 6 h stimulation period, reduced sleep by 48.2%, which is consistent with the sleep loss observed in the AIP model. Many other laboratories have produced similar pain models,^[^ ^31^, ^32^, ^33^, ^71^, ^72^, ^73^ ^]^ but only a few have objectively analyzed them for sleep and sleep fragmentation.^[^ ^5^, ^7^ ^]^ Reliable and quantifiable pain assessment is crucial to its diagnosis and effective management, which can be done with specific biomarkers. Our data from both pain models show that we can reliably use objective criteria of sleep loss and fragmentation as a tool to detect how manipulations of the PBel^CGRP^ neurons and their terminals affect sleep‐wake, in addition to comparing the site‐specific efficacy of blocking their action on pain‐induced sleep loss.

Our results of genetically ablating PBel^CGRP^ neurons show that such deletions reversed AIP‐induced sleep loss by 87%, reversed sleep fragmentation, prevented the increase in slow wave perception, and increased spindle density, suggesting that there was a significant decrease in nociceptive signals that drive circuits regulating wakefulness.^[^ ^69^, ^70^ ^]^ Similarly, optogenetic silencing of PBel^CGRP^ neurons (using JAWS) before activation of the CGRP nociceptors to induce pain‐like effects also prevented short latency awakenings and completely reversed the opto‐pain‐induced sleep loss. Results from the AIP and opto‐pain models suggest that PBel^CGRP^ neurons are the key relay through which acute peripheral injury drives wakefulness from NREM sleep.

Our data also corroborate the anatomical findings that the PB receives spinal nociceptive‐specific projections from the lamina I^[^ ^27^ ^]^ and is activated by a wide range of nociceptive stimuli.^[^ ^25^, ^26^, ^27^, ^28^, ^74^ ^]^ Activation of excitatory neurons in the lateral PB of the mice caused place aversion, while their inhibition reduced pain‐related responses.^[^ ^17^ ^]^ In particular, the PBel^CGRP^ neurons are involved in several components of pain behavior, including escaping from a noxious stimulus and forming aversive memory. Studies have shown that mice with ablation of PBel^CGRP^ neurons have attenuated reactions to a noxious heat source and do not develop conditioned place aversion, while their activation promotes aversion.^[^ ^29^ ^]^ Additionally, neuropathic and inflammatory‐induced pain produced amplified responses in these neurons, which lasted for 5 weeks, parallel to the increased pain metric measurements.^[^ ^30^ ^]^ A recent study also showed that ablation of the PBel^CGRP^ neurons attenuated sleep fragmentation in a chronic neuropathic pain model;^[^ ^5^ ^]^ however, pharmacological blockade of skin sensory fibers was ineffective, indicating that even in chronic pain, pain is relayed through the PBel^CGRP^ neurons to cause brief arousals.^[^ ^5^ ^]^ Similarly, in our study, mice with either genetic deletion or optogenetic silencing of PBel^CGRP^ neurons had almost no sleep loss in response to a pain stimulus, suggesting that blocking this node blocks pain‐induced disruption of normal sleep. However, this is not due to their sleep‐promoting effect, as inhibition alone without a pain stimulus did not increase or promote sleep. Our data also reinforce the results from our previous study, which showed that these neurons regulate cortical arousals in response to other aversive stimuli, such as hypercapnia (brief periods of breathing pauses, or apnea, during sleep), through their projections to the CeA and SI‐BF.

Next, we dissected the underlying neural pathways by conducting optogenetic inhibition of four different terminal sites targeted by the PBel^CGRP^ neurons to understand which of them are critical for driving cortical arousals from sleep in response to activation of nociceptive primary afferents in the AIP model. Optogenetic blocking of the terminal sites followed the effects order of SI‐BF > CeA > BNST > LH for reversal of AIP‐induced sleep loss and fragmentation. As shown by others,^[^ ^16^, ^25^, ^34^ ^]^ our results also confirm the importance of the PBel^CGRP^ → CeA pathway in pain‐induced sleep disturbance and, in addition, identified the novel roles of two other projection sites, SI‐BF and BNST, which have long been associated with the mediation of arousal^[^ ^9^, ^10^, ^14^, ^75^, ^76^ ^]^ and stress‐related fear and anxiety.^[^ ^12^, ^42^, ^77^, ^78^ ^]^

Additionally, our data is supported by a recent study, which shows that cholinergic neurons in the nucleus basalis area of the BF that receive projections from the PB are overactive in a mouse model of neuropathic pain.^[^ ^79^ ^]^ Similarly, other studies suggest that the BNST mediates nocifensive responses to pain.^[^ ^16^ ^]^ However, in contrast to some of the previous studies^[^ ^12^, ^16^, ^80^, ^81^, ^82^ ^]^ that implicate the PBel→ LH pathway in pain‐induced adverse behaviors, we did not observe any significant role of this pathway in mediating pain‐induced sleep disturbances, which could possibly be attributed to targeting sparse projections of the PBel^CGRP^ neurons in the LH. These results are also in parallel with our earlier study, where we investigated the arousal response to hypercapnia^[^ ^18^ ^]^ and found that inhibition of the PBel^CGRP^ terminal field in the BF had the most profound effects, while inputs to the LH made little or no contribution.

Lastly, pharmacological blocking at either the SI‐BF or CeA using CGRP or NMDA receptor antagonists had proportionally similar effects on reversing AIP‐induced sleep loss. PBel^CGRP^ neurons are glutamatergic, so their activation in response to pain causes the release of both glutamate and CGRP, which then act on NMDA and CGRP receptors at their terminal sites. Furthermore, our findings align with an earlier study that investigated the lateral PB (LPB)→CeA circuits in slice recordings and showed that synaptic transmission is mediated by glutamate.^[^ ^19^, ^35^ ^]^ However, the fibers from the LPB also release CGRP in the CeA, where it potentiates synaptic NMDA‐R function,^[^ ^12^, ^35^ ^]^ and could have a potent impact on the strengthening of the nociception‐emotion‐sleep link in persistent pain. NMDA or CGRP blockers injected in SI‐BF selectively blocked the acute‐pain‐induced sleep disturbances but did not alter normal spontaneous sleep, analogous to an earlier study where neither cell nor vesicular glutamate transporter (Vglut2) gene deletions in the LPB changed spontaneous sleep.^[^ ^83^ ^]^ A recent study also identified the role of PBel^CGRP^ in chronic neuropathic pain‐induced microarousals.^[^ ^5^ ^]^ Thus, our results help identify the precise role of the PBel^CGRP^ pathways that cause awakenings in response to nociceptor activation during acute tonic pain, which may be clinically relevant in preventing pain‐induced sleep disturbances without affecting normal spontaneous sleep.

In conclusion, our findings establish a neural network that links pain and sleep disturbances. This process is mediated by the transmission of nociceptive signals through the spino‐parabrachial pathways, which converge on the PBel^CGRP^ node in the dorsal pons.^[^ ^27^ ^]^ The glutamatergic PBel^CGRP^ neurons play a role in regulating these sleep disturbances by projecting to key forebrain arousal areas, namely the substantia innominata of the basal forebrain (SI‐BF), and the central amygdala (CeA), in a similar proportion. These neurons also project to the bed nucleus of the stria terminalis (BNST). At these target sites, their effects are mediated by action on NMDA and CGRP receptors. These results suggest that these sites could be targeted for the development of non‐addictive analgesics, utilizing compounds that act on non‐opioid receptors found in these areas. Recently, CGRP receptor blockers (known as gepants) and monoclonal antibodies targeting CGRP receptors have been shown to treat headaches and migraines^[^ ^84^, ^85^, ^86^ ^]^ effectively; however, the central sites of action for these treatments remain unclear. It is unlikely that they cross the blood‐brain barrier and target CGRP receptors in the BF or CeA at concentrations that allow them to alter neuronal responses.^[^ ^87^, ^88^, ^89^ ^]^ However, whether high central CGRP‐receptor antagonism may provide any additional therapeutic benefit or help the non‐responders^[^ ^90^ ^]^ remains to be understood. Here, it is also important to point out that injections of neither BIBN nor AP5 alone in the SI‐BF (Figure 6c) altered sleep‐wake behavior. Thus, the reported adverse effects like, fatigue caused by CGRP monoclonal antibodies and CGRP antagonists,^[^ ^91^, ^92^ ^]^ is likely caused by their peripheral site of action, i.e., due to vascular constriction by these drugs that may lead to reduced oxygen levels in multiple peripheral organs, and not due to inhibiting the CGRP signaling in the CNS.

A few limitations of our study are that it did not include chronic pain models or investigate female mice, which have higher pain sensitivity,^[^ ^93^ ^]^ that varies with the estrous state.^[^ ^94^ ^]^ However, similar to our results, a recently published study using the chronic neuropathic pain model found that genetic ablation of PBel^CGRP^ neurons also attenuated sleep fragmentation,^[^ ^5^ ^]^ suggesting that PBel^CGRP^ neurons are equally important in chronic pain and may also drive nociplasticity associated with chronic pain.^[^ ^23^ ^]^ Further studies are required in the future to understand the circuits underlying central sensitization evoked by chronic pain. Based on the existing literature, sex‐based biological differences in pain processing that contribute to heightened pain perception and sensitivity in females may possibly be attributed to the differences in CGRP‐mediated synaptic plasticity^[^ ^95^, ^96^ ^]^ in the PB→CeA pathways. This suggests that the affective pain processing is being differentially modulated in females, but requires further investigations into the cell‐specific details that potentiate these effects.

Prolonged sleep disturbances alter pain sensitivity, decrease the effectiveness of opioid analgesics, and delay recovery from pain,^[^ ^6^, ^7^, ^59^, ^60^, ^61^ ^]^ while pain directly contributes to sleep disruption.^[^ ^5^, ^28^, ^97^, ^98^, ^99^ ^]^ This bidirectional link between sleep and pain potentially suggests involvement of the common anatomical pathways, making the assessment of sleep disturbances in both acute and chronic pain a useful biomarker for evaluating the effectiveness of therapeutic interventions aimed at managing pain and enhancing patients' quality of life.

Future research should adopt integrative approaches that combine objective assessments of both sleep and pain, along with utilizing existing or novel interventions designed to specifically target and investigate common anatomical pathways that can improve the reduced quality of life (i.e., disease burden) in patients with chronic and acute pain who experience sleep disruptions. Additionally, future studies could leverage innovative tools, such as a spatially resolved transcriptional atlas,^[^ ^100^ ^]^ to identify specific cell types and unique receptor types located at key points along these wake‐pain‐relay pathways that could be targeted to alleviate both sleep disturbances and pain with minimal addiction liability.

## 4 Experimental Section

### Animals

Male mice (CGRP‐creER, CGRP‐ChR2, their wildtype littermates, and C57BL/6J mice) were bred in the Animal Research Facility using a heterozygote breeding scheme to ensure true littermates as control animals. All mice were backcrossed to the C57BL6 strain for at least six generations and were outbred to C57BL/6J mice obtained from Jackson Labs every three generations. Mice were group‐housed until implantation, then singly housed with ad libitum access to water and food, ambient temperature of 21–23°C, humidity levels between 40% and 60%, and a 12‐h light/dark cycle. Wildtype littermates were randomly assigned to experimental groups. All animal procedures and protocols were approved by the Beth Israel Deaconess Medical Center Institutional Animal Care and Use Committee (Protocol approval #‐ 032‐2020‐23) and meet National Institutes of Health standards.

### Validation of Mice

CGRP‐ChR2 were validated by double immunolabeling for CGRP and mCherry (co‐expressing ChR2) (Figure S2, Supporting Information), where the neurons in PB that were CGRP were labeled for ChR2, and similar labeling was also seen in terminal sites, e.g., in the CeA and BNST, suggesting reliable expression of ChR2 in the CGRP neurons and terminals in the brain (Figure S2a,b, Supporting Information). In addition, we also confirmed that the primary afferent CGRP neurons in the DRG and the CGRP nerve endings in the skin of the hind foot express ChR2 (Figure S2c,d, Supporting Information) as well. CGRP‐creER mice had previously been validated for the eutopic presence of the enzyme cre‐recombinase in the CGRP‐expressing neurons.^[^ ^18^ ^]^

### Viral Vectors

AAV‐Flex‐DTA was previously validated for specific cre‐dependent ablation of PBel^CGRP^ neurons in the CGRP‐creER and DR‐serotonergic neurons in the Sert‐Cre mice.^[^ ^18^, ^21^, ^37^ ^]^ AAV8‐hsyn‐Flex‐JAWS‐GFP (obtained from University of North Carolina Vector Core, Lot # AV6640B, 3.2 × 10^12^ virus molecules mL^−1^ dialyzed with 350 mm NaCl & 5% D‐sorbitol in PBS) had also been verified by the group and others for causing effective inhibition by red laser (635 nm) / LED light (Figure 2c). Additionally, pAAV5‐hsyn1‐SIO‐eOPN3‐mScarlet‐WPRE (Addgene, Catalog # 125713‐AAV5, Lot # v128152) was used, which others have also validated for causing effective inhibition with blue (460 nm) light.^[^ ^38^, ^39^, ^40^ ^]^

### Surgery—For Genetic Deletion, Optogenetic Silencing, and Opto‐Terminal Inhibition Experiments

Under surgical anesthesia, male CGRP‐creER or CGRP‐ChR2 transgenic mice aged 6–12 weeks and their wild‐type littermate (as controls) received micro‐brain injections (320 nl) of a viral‐vector (AAV‐Flex‐DTA, AAV8‐hsyn‐Flex‐JAWS‐GFP, or pAAV‐hSyn1‐SIO‐eOPN3‐mScarlet) targeted bilaterally to the PB area (AP: −5.2 to −5.3 mm; DV−2.7 to −2.8 mm; ML: ±1.3 to 1.33 mm). To induce cre‐expression, mice received intraperitoneal tamoxifen injections (75 mg kg^−1^ diluted in corn oil) for five consecutive days concurrent with their corresponding micro‐brain injections. After 4 weeks of recovery, all injected mice were implanted with EEG and EMG electrodes. Mice that received AAV‐Flex‐JAWS were also implanted with bilateral optical fibers (Doric Lenses, dual fiber optic cannulas, 200 µm core, 2.6 mm distance between fibers, 3–5 mm length, TFC_200/245‐0.37_5 mm_TS2.6_FLT) targeted to the PB (same coordinates as above), and a NeuroLux micro‐LED (u‐iLED) inserted sub‐dermally with the flexible micro‐fiber directed to the footpad (hind paw) and secured under the skin with sutures (NeuroLux implantable devices, 473 nm, 4 mm). In addition to EEG/EMG electrodes, mice that received AAV‐Flex‐eOPN3 were also implanted with bilateral optical fibers targeted to one of the terminals sites, SI‐BF (AP: −0.15 mm; DV −4.5 mm; ML: ±1.2 mm), BNST (AP: +0.3 mm; DV −4.0 mm; ML: ±1.0 mm), CeA (AP: −1.35 mm; DV −4.4 to −4.6 mm; ML: ±2.75 mm), or LH (AP: −1.4 mm; DV −5.0 mm; ML: ±1.4 to 1.5 mm). Mice were singly housed following implantation and allowed 7–10 days of recovery.

### Surgery—For Pharmacological Blocking Experiments

Under surgical anesthesia, male wild‐type C57BL/6J mice aged 6–12 weeks were implanted with EEG and EMG electrodes as well as cannulas targeted to either SI‐BF (AP: −0.15 mm; DV −4.5 mm; ML: ±1.2 mm) or CeA (AP: −1.35 mm; DV −4.4 to −4.6 mm; ML: ±2.75 mm). Mice were singly housed following implantation and allowed 7–10 days of recovery.

### Data Acquisition

Following surgical implantation, mice were habituated to the recording chambers, and requisite tethering equipment was used to record EEG and EMG for 7–10 days. Additionally, mice recorded for optogenetic silencing and opto‐inhibition were allowed at least 2 days to habituate to the fiber optic cables. All recordings were acquired at least 5 weeks post‐injection of viral vectors to ensure optimal expression and started at ZT2. Each mouse was connected to a preamplifier, which in turn was connected to a commutator, and then to a 8242‐3‐channel analog adapter, all from Pinnacle Technology. The analog adapters were connected to a data acquisition console from Cambridge Electronic Design (Cambridge, United Kingdom) and recorded using their corresponding software, Spike2.

### Sleep Data Acquisition for AIP Induced Sleep Loss in Mice with and without Genetic Deletion of the PBel^CGRP^ Neurons

Recording all the treatment groups for 24 h of the EEG and EMG recordings after the following conditions were conducted in a random order with 7 days recovery between each: 1) no intervention (baseline), 2) following 25 µl 0.9% saline injection in the hind limb or 3) following 25 µl 5% formalin sub‐dermally in the hind limb (AIP).

### In Vitro Electrophysiological Recordings of the PBel^CGRP^ Neurons that Expressed JAWS for Validating Silencing by Laser

For in vitro electrophysiological recordings, CGRP‐creER mice (*n* = 3) were used. A total of 200 nl of AAV‐FLEX‐JAWS‐GFP was injected into the PB of the CGRP‐creER mice, using the coordinates described above. Three to four weeks after AAV injections, the mice were deeply anesthetized with isoflurane (5% in oxygen) via inhalation and transcardially perfused with ice‐cold ACSF (N‐methyl‐D‐glucamine, NMDG‐based solution, as described below). The brains were then quickly removed and coronally sectioned at 250 µm thickness in ice‐cold NMDG‐based ACSF using a vibrating microtome (VT1200S, Leica). Slices containing the PB were first incubated in the NMDG‐based ACSF for 10 min at 37 °C, then transferred them to a holding chamber at 37 °C containing Na‐based ACSF for an additional 10 min. The brain slices were allowed to gradually return to room temperature (≈1 h) before recording. Brain slices were submerged in a recording chamber and perfused with Na‐based ACSF (1–1.5 mL min^−1^, described below). PBel^CGRP^ neurons that expressed JAWS‐GFP (identified by green fluorescence) and those in the PB that did not express JAWS‐GFP as controls (no fluorescence) were recorded, using a combination of fluorescence and infrared differential interference contrast microscopy (IRDIC). Recordings were made using a fixed‐stage upright microscope (BX51WI, Olympus America) equipped with a Nomarski water immersion lens (Olympus 40X / 0.8 NAW) and an IR‐sensitive CCD camera (ORCA‐ER, Hamamatsu), with real‐time imaging acquired using Micro‐Manager software. Neurons were recorded in current‐clamp configurations using a Multiclamp 700B amplifier (Molecular Devices), a Digidata 1322A interface, and Clampex 9.0 software (Molecular Devices). Neurons showing more than a 10% change in input resistance over the course of the recording were excluded from the analysis. JAWS‐GFP expressing neurons in PB were photo‐inhibited using full‐field light openings (≈1.8 mW mm^2−1^, 1 mm beam width) from a 445 mW LUXEON light‐emitting diode (#M595L3; Thorlabs, Newton, NJ, USA) coupled to the epifluorescence pathway of the microscope. PB neurons were recorded before, during, and after 20 s‐long continuous light activation. Recordings were made using K‐gluconate‐based pipette solution, in whole‐cell current clamp mode (described below). In all the recordings, 0.5% biocytin were added to the pipette solution to mark the recorded neurons.^[^ ^101^ ^]^ After in vitro recordings, to label the recorded neurons filled with biocytin, the recorded slices were fixed in 10% buffered formalin, washed them, and incubated them overnight in streptavidin‐conjugated Alexa Fluor 555 (1:500; Cat#: S32351; Invitrogen, Thermo Fisher Scientific Waltham, MA). Images were acquired using a Leica Stellaris 5 confocal microscope using a 63X oil immersion objective.^[^ ^102^ ^]^

### Solutions for Electrophysiological Recordings

NMDG‐based ACSF solution containing (in mM): 100 NMDG, 2.5 KCl, 1.24 NaH_2_PO_4_, 30 NaHCO_3_, 25 glucose, 20 HEPES, 2 thiourea, 5 Na‐L‐ascorbate, 3 Na‐pyruvate, 0.5 CaCl_2_, 10 MgSO_4_ (pH 7.3: 95% O_2_ and 5% CO_2_; 310–320 mOsm). Na‐based ACSF solution contained (in mM): 120 NaCl, 2.5 KCl, 1.3 MgCl_2_, 10 glucose, 26 NaHCO_3_, 1.24 NaH_2_PO_4_, 4 CaCl_2_, 2 thiourea, 1 Na‐L‐ascorbate, 3 Na‐pyruvate (pH 7.3–7.4 in 95% O_2_ and 5% CO_2_; 310–320 mOsm). K‐gluconate‐based pipette solution containing (in mM): 120 K‐Gluconate, 10 KCl, 3 MgCl2, 10 HEPES, 2.5 K‐ATP, 0.5 Na‐GTP (pH 7.2 adjusted with KOH; 280 mm). All other chemicals were purchased from Fisher Scientific (Waltham, MA) or Sigma–Aldrich (Saint Louis, MO).

### Sleep Data Acquisition with Optogenetic Silencing of the PBel^CGRP^ Neurons and Acute Opto‐Pain Stimulus

All treatment groups were recorded for the 24 h of EEG and EMG recordings under the following conditions, which were conducted in random order with 7 days of recovery between each treatment: 1) no intervention (baseline), 2) Opto‐pain (10 Hz, 10 ms, 4 s “ON” every 5 min from ZT1‐ZT10, 473 nm) without inhibition of PBel^CGRP^ neurons, 3) Opto‐pain + opto‐inhibition (20 s‐ON and 5 min‐OFF from ZT1‐ZT10, using red laser‐ 635 nm), where laser inhibition preceded the opto‐pain stimulation by 10 s for each trial.

Opto‐pain stimulation was triggered with the NeuroLux Optogenetic Smart System via the PDC box and auto‐tuner with external loop antennae, which were placed around the recording chambers (NeuroLux, Northfield, IL, USA). This allowed for activation of the radio frequency field generated by the antennae to trigger the implanted NeuroLux device, causing the emission of 473 nm laser light within the device implanted in the hindlimb (for which the µLED was directed to the footpad) that optogenetically stimulated CGRP peripheral receptors, which caused local pain sensation. The optogenetic silencing of PBel^CGRP‐JAWS^ neurons was triggered via a stimulation protocol executed by Spike2, which also recorded EEG and EMG for offline analysis of sleep‐wake behavioral states. The red laser light was transmitted through a fiber optic splitter for bilateral stimulation (TM105FS1B, Thorlabs, NJ) to fiber optic cables (1.5 m length, 200 µm diameter core, Doric Lenses, Quebec, QC, Canada). The laser power emitted from the end of the fiber optic cables was measured before and after recordings to ensure it was within a range of 8–10 mW, though this was likely a higher estimate due to light lost at the connection of the fiber optic cable and implant. Laser stimulations at similar power had previously used for comparable and longer durations (continuous stimulation for 1 min every 5 min),^[^ ^18^, ^37^ ^]^ yet no tissue damage had been observed, as was also the case for the present experiments.

### Sleep Data Acquisition with Opto‐Inhibition of the PBel^CGRP^ Terminal Sites with and without AIP

All groups of mice undergo 24 h of EEG and EMG recordings, under the following conditions that were conducted in random order with 7 days of recovery between each: 1) no intervention (baseline), 2) following 25 µl 0.9% saline injection in the hind limb, 3) following 25 µl 5% formalin (AIP) in the hind limb 4) following saline with opto‐inhibition (4 s on, 4 s off from ZT2‐ZT8, using LED of 460 nm wavelength), 5) following AIP with opto‐inhibition (4 s on, 4 s off from ZT2‐ZT8). LED light stimulation was triggered ON via a script in Spike2, which was used for simultaneous sleep recordings. LED (460 nm) light was transmitted (Prizmatix, Holon, Israel) through a fiber optic coupler (Fiber Optic Couplers, 1×2 FOC /200u/L3.0M/1.25/FC, Splitting Ratio: 50:50.0.37 N.A., Prizmatix) that split the beam into two for bilateral stimulation (4 s on, 4 s off from ZT2‐ZT8, using LED of 460 nm wavelength). LED power was measured before and after recordings to ensure it was within the 6 mW range, although this was likely a higher estimate, considering some light was lost at the connection of the fiber optic cable and implant.

### Sleep Data Acquisition with Pharmacological Blocking of the Terminal Sites

All mice implanted with EEG/EMG and guide cannulas targeting either SI‐BF or CeA were recorded for 24 h of EEG and EMG after the following conditions that were conducted in varying order, all with 7 days of recovery in between each: 1) no intervention (baseline), 2) following 25 µl 0.9% saline injection in the hind limb, 3) following 25 µl 5% formalin (AIP) in the hind limb, 4) following saline injection after bilateral injections of CGRP‐receptor blocker (80 µg µl^−1^ dissolved in 1% DMSO) in either SI‐BF or CeA, 5) following AIP after CGRP‐receptor blocking, 6) following saline injection after bilateral injections of NMDA‐receptor blocker (40 µg µl^−1^ dissolved in 0.9% saline) in either SI‐BF or CeA, 7) following AIP after NMDA‐receptor blocking, 8) following CGRP‐receptor blocking, and 9) following NMDA‐receptor blocking. Receptor blockers were delivered using the injector cannula (33G, Pinnacle Technologies, Roanoke, VA) directed via pre‐implanted guide cannulas (C235GS‐5‐2.0/SPC Guide 39622 26GA DBL 5 mm PED, P1 Technologies, Roanoke, VA) at a rate of 3.3 nl s^−1^ for a total volume of 200 nl using PE10 tubing (Fisher Scientific) connected to a Hamilton syringe (Catalog # 80330, 701RN 10 µl SYR 26s/2″/2, Lot # 1055245). After drug injections were completed within 1–2 min, the injector cannulas were left in place to minimize post‐injection handling.

### Data Analysis

*Sleep analysis‐* EEG and EMG recordings were converted from Spike2 to SleepSign software (Kissei Comtec, Matsumoto, Nagano, Japan) and analyzed in 4‐s epochs for sleep state (wake, non‐REM, and REM). The files were first auto‐scored using an algorithm based on EMG power, EEG delta, and EEG theta power, and then manually scored by researchers who were blind to the treatment groups (NL, RLS, SS, JDL, SK). EEG power spectral densities (PSDs) were computed using the multi‐taper method (Chronux toolbox; http://chronux.org) as described previously.^[^ ^103^ ^]^ Sleep spindles were detected using an automated algorithm (MATLAB) that had been previously validated and published.^[^ ^104^, ^105^ ^]^ Briefly, EEG data were band‐pass filtered (9–15 Hz, Butterworth Filter), and the root‐mean‐squared (RMS) power was calculated to provide an upper envelope of the data. The RMS data was then exponentially transformed to further accentuate spindle‐generated signals over the baseline. Putative spindle peaks (example in Figure S1a, Supporting Information) were identified in transformed data via crossing of an upper‐threshold value, set as 3.5x the mean RMS EEG power across all states for each mouse. Additional detection criteria included a minimum duration of 0.5 s, based on the crossing of a lower threshold set at 1.2x mean RMS power, and a minimum inter‐event interval of 0.5 s. This automated spindle detection algorithm had been rigorously tested and compared with manual spindle detection.^[^ ^104^, ^105^ ^]^

### Statistical Analysis—For Behavioral Sleep States Comparison

The quantitative (percent time, sleep bout number, and durations) or qualitative (EEG power in sleep‐wake states and spindle density) sleep data were statistically compared and plotted using the SigmaPlot software (Systat Software Inc., version 14.5). Figures were composed in Photoshop (Adobe) software. All data presented in figures were represented as mean ± SEM, where “n*”* refers to the number of animals. All treatment groups were compared using either one‐way or two‐way ANOVA followed by the Holm‐Sidak method for multiple comparisons *post‐hoc* test. Values showing *p* < 0.05 were considered significant.

### Statistical Analysis—For In Vitro Electrophysiological Recordings

The recorded data were analysed using Clampfit 10 (Molecular Devices) software. Figures were generated using Igor Pro version 6 (WaveMetrics), Prism 7 (GraphPad, La Jolla, CA), and Photoshop (Adobe) software. Membrane potential changes were calculated by comparing values before, during, and after light stimulation. Data were represented as mean ± SEM, and *n* refers to the number of cells, and compared group means using one‐way ANOVA repeated measures followed by Bonferroni's multiple comparisons *post‐hoc* test. Values showing *p* < 0.05 were considered significant.

### Histology Remove the strikethrough words

After all in vivo recordings were completed, mice were deeply anesthetized and transcardially perfused initially with 0.9% saline, followed by 10% buffered formalin. Subsequently, brains were collected and stored in 30% sucrose in 0.1 M phosphate buffer with 0.02% sodium azide overnight before they were sectioned with a freezing microtome in four series of 30 µm sections. Sections of each brain were stored in 0.1 M phosphate buffer and 0.02% sodium azide at 4°.

### Immunohistochemistry

For mice recorded in genetic deletion, optogenetic silencing, or opto‐inhibition experiments, one series of sections from each brain was immunolabeled for the proteins using the primary and secondary antibodies described in Table **1**. For the genetic deletion experiments, brain sections from the CGRP‐creER or WT mice that received brain injections of AAV‐Flex‐DTA were immunolabeled for dsRed and CGRP (Figure 1d) to analyze and confirm PBel^CGRP^ neuronal deletions. For the optogenetic silencing experiments, CGRP‐ChR2 and WT mice that received brain injections of AAV8‐hsyn‐Flex‐JAWS‐GFP were immuno‐stained for GFP and CGRP (Figure 2d) to confirm PBel^CGRP‐JAWS^ transfection and optical fiber tract location. Lastly, CGRP‐creER and WT littermates that were used in opto‐inhibition recordings that received brain injections of AAV‐hSyn1‐SIO‐eOPN3‐mScarlet were immuno‐labeled for dsRed (Figure 4) to confirm if the optical fibers at the terminal sites targeted the transfected terminal fields.

**Table 1:** Details of antibodies used for immunohistochemistry.

Antigen	Host	Dilution	Source	Immunogen	Validation	Secondary Incubation	Dilution
CGRP	Goat	1:1000	Abcam Catalog # Ab36001 Lot # GR253001‐9	–	No staining with genetic deletion of CGRP neurons	Biotinylated donkey anti‐goat, followed by Streptavidin‐Cy2	1:200
GFP	Chicken	1:1000	Invitrogen Catalog # A10262 Lot # 2738236	H3‐GFP construct transfected in HEK‐293E cells	Absence of staining confirmed in wild‐type and uninjected mice	Biotinylated anti‐chicken Alexa Fluor 488 (Invitrogen)	1:200
DsRed	Rabbit	1:1000	Takara Bio Catalog # 632496 Lot # 1612022	DsRed‐Express, a variant of Discosoma sp. red fluorescent protein	Absence of staining confirmed in wild type and uninjected mice	Biotinylated anti‐rabbit Alexa Fluor 555 (Invitrogen)	1:200

Standard immunostaining protocols previously used by the group were followed.^[^ ^18^, ^21^ ^]^ All sections were incubated overnight in the respective primary antibody (details in Table 1) diluted in phosphate‐buffered saline with Triton‐X‐100 (0.25%) and sodium azide (0.02%). On the second day, sections were incubated with the secondary antibody for 2 h (Table 1), which was either biotinylated or labeled with a fluorescent tag (Alexa Fluor‐488, Alexa Fluor‐555, or Cy5). Those incubated in biotinylated secondary were then incubated with a streptavidin‐labeled fluorescent (Alexa Fluor‐ 555 or Cy5). Finally, sections were mounted on Super‐Frost Plus slides (Fisher Scientific, Pittsburgh, PA), cover‐slipped with Dako fluorescent mounting medium (Agilent Technologies, Santa Clara, CA), and then scanned using either an Olympus SlideView VS200 slide scanner or a Leica Stellaris 5 confocal microscope.

For the mice recorded in the pharmacological blocking experiments, one series of sections from each brain were mounted on Super‐Frost Plus slides (Fisher Scientific, Pittsburgh, PA), dried overnight at room temperature, and then stained for Nissl using the following protocol. Slides were first washed in distilled water, stained for 1 min, then in 0.1% thionin diluted in distilled water, then dehydrated in varying concentrations of EtOH, cleared in Xylene, cover‐slipped using Permaslip (Alban Scientific, St. Louis, MO), and then scanned using the Olympus SlideView VS200 slide scanner.

### Dissection and Immunostaining of Lumbar Dorsal Root Ganglia and Nerve Endings in the Foot Pad for CGRP and ChR2

After perfusing CGRP‐ChR2 mice (*n* = 3) as per the method described above, their hind limbs and spinal column were harvested and fixed overnight at 4 °C in Zamboni fixative (Newcomers Supply, Middleton, WI) for 24–72 h. Skin from the hind footpad was dissected out and washed in phosphate buffer as per the method described in Liu et al., 2017.^[^ ^106^ ^]^ The spinal column was further dissected for isolation of the L3‐L4 DRG from the lumbar enlargement of the spine as described in Sleigh et al., 2016.^[^ ^107^ ^]^ Post dissection, both the tissues, the skin from the footpad, and the L3‐L4 DRGs were transferred to cryoprotectant (30% sucrose) solution in separate labeled tubes for 24 h, following which they were embedded in Tissue‐Tek OCT compound as blocks for sectioning at 30 µm in a cryostat, where the cut sections were directly mounted on slides. The slide‐mounted sections followed similar standard immunohistochemical procedures as described above, using antibodies against CGRP and ChR2 to identify co‐labeling, cover‐slipped with Dako fluorescent mounting medium (Agilent Technologies, Santa Clara, CA), and then scanned using a Leica Stellaris 5 confocal microscope (Figure S2, Supporting Information).

## Conflict of Interest

The authors declare no conflict of interest.

## Author Contributions

N.L. designed the experiments, collected and analyzed data, and wrote the manuscript. R.D.L., E.R., and E.A. collected and analyzed in vitro data. R.L.S. collected and analyzed data. R.C.T., S.S., N.G., and J.D.L. analyzed data and processed brain tissue. S.S.B. managed the mouse breeding program. R.B. contributed to manuscript preparation and provided critical insights. A.M.C. dissected and stained the dorsal root ganglia. S.T. and E.E.G. performed power and spindle analysis and contributed to manuscript writing. S.K. conceptualized and designed the experiments, collected and analyzed data, and wrote the manuscript.

## Supporting information



Supporting Information

## Data Availability

The source data file has been provided with this paper. Additional data generated to support the findings of this study are also available from the corresponding author on request.
