# Supplementary material for: Calcitonin Gene‐Related Peptide (CGRP)‐Expressing Neurons in the External Lateral Parabrachial Area Regulate Pain‐Induced Sleep Disturbances
Source: Adv Sci (Weinh). 2025 Jun 29;12(35):e00325. doi: 10.1002/advs.202500325 (PMC12463127; doi:10.1002/advs.202500325)
Supplement: Supplementary file 1 — Supporting Information [file ADVS-12-e00325-s001.pdf]

ADVANCED SCIENCE
Open Access

Supporting Information

for *Adv. Sci.*, DOI 10.1002/advs.202500325

Calcitonin Gene-Related Peptide (CGRP)-Expressing Neurons in the External Lateral Parabrachial Area Regulate Pain-Induced Sleep Disturbances

*Nicole Lynch, Roberto De Luca, Richard L Spinieli, Enrico Rillosi, Renner C Thomas, Samuel Sailesh, Nishta Gangeddula, Janayna D Lima, Sathyajit S Bandaru, Elda Arrigoni, Agustin Melo-Carrillo, Rami Burstein, Stephen Thankachan and Satvinder Kaur**

Calcitonin Gene-Related Peptide (CGRP)-Expressing Neurons in the External Lateral Parabrachial Area Regulate Pain-Induced Sleep Disturbances

Nicole Lynch[1], Roberto De Luca[1], Richard L Spinieli[1], Enrico Rillosi[1], Renner C Thomas[1], Samuel Sailesh[1], Nishta Gangeddula[1], Janayna D Lima[1], Sathyajit Bandaru[1], Elda Arrigoni[1], Agustin Melo-Carrillo [1,3], Rami Burstein[1,3], Stephen Thankachan[2] and Satvinder Kaur[1]*

Supplementary Figures

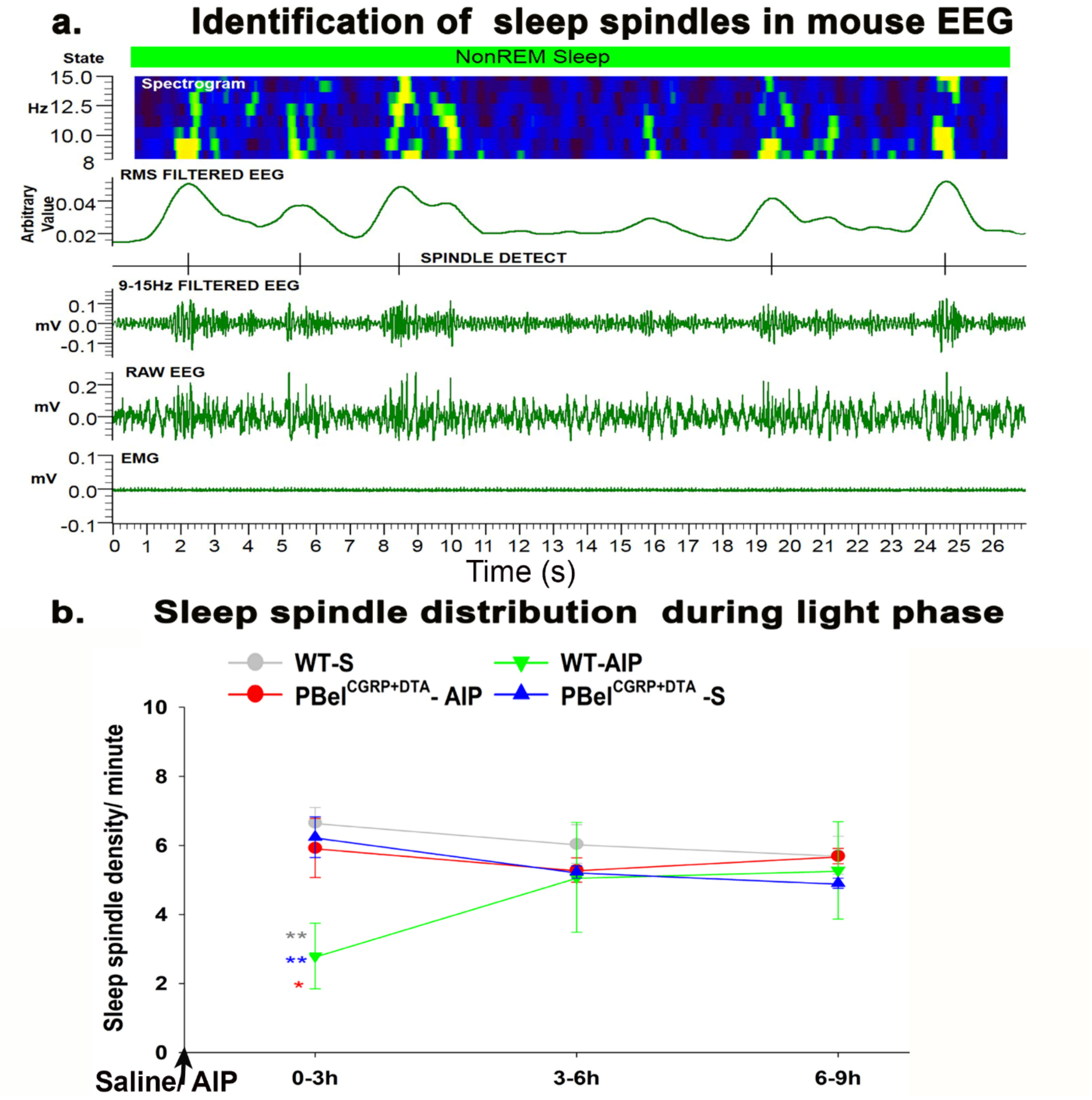
a. Identification of sleep spindles in mouse EEG
State
NonREM Sleep
Spectrogram
Hz
15.0
12.5
10.0
8
RMS FILTERED EEG
Arbitrary Value
0.04
0.02
SPINDLE DETECT
9-15Hz FILTERED EEG
mV
0.1
0.0
-0.1
RAW EEG
mV
0.2
0.0
EMG
mV
0.1
0.0
-0.1
0 1 2 3 4 5 6 7 8 9 10 11 12 13 14 15 16 17 18 19 20 21 22 23 24 25 26
Time (s)
b. Sleep spindle distribution during light phase
WT-S
WT-AIP
PBel CGRP+DTA - AIP
PBel CGRP+DTA -S
Sleep spindle density/ minute
10
8
6
4
2
0
**
**
*
Saline/ AIP
0-3h
3-6h
6-9h

Figure S1

Supplementary Figure S1: *Representations of sleep spindles detection and their distributions across the light phase:*

a) Representative EEG trace, EMG trace, sleep spindle detection trace, along with filtered EEG, and spectrogram of EEG power frequencies for 30s of data

b) Graph showing sleep spindle density (spindles/min, mean ± SEM) in 3h bins over 9h post-injection in the following groups: WT mice with saline injection (WT-S; n=4), WT mice with formalin injection (**WT-AIP**; n=4), **PBel$^{\mathbf{CGRP\text{-}DTA}}$** mice with saline injection (**PBel$^{\mathbf{CGRP\text{-}DTA}}$**; n=4), and **PBel$^{\mathbf{CGRP\text{-}DTA}}$** mice with formalin injection (**PBel$^{\mathbf{CGRP\text{-}DTA}}$-AIP**; n=4). Groups were compared using a two-way (treatment x time) ANOVA, followed by the Holms-Sidak method for multiple comparisons, where **- $P< 0.001$; *- $P< 0.05$. The color of the asterisk represents the group being compared to WT-AIP. Exact *P* values for each comparison are mentioned in detail in the results section.

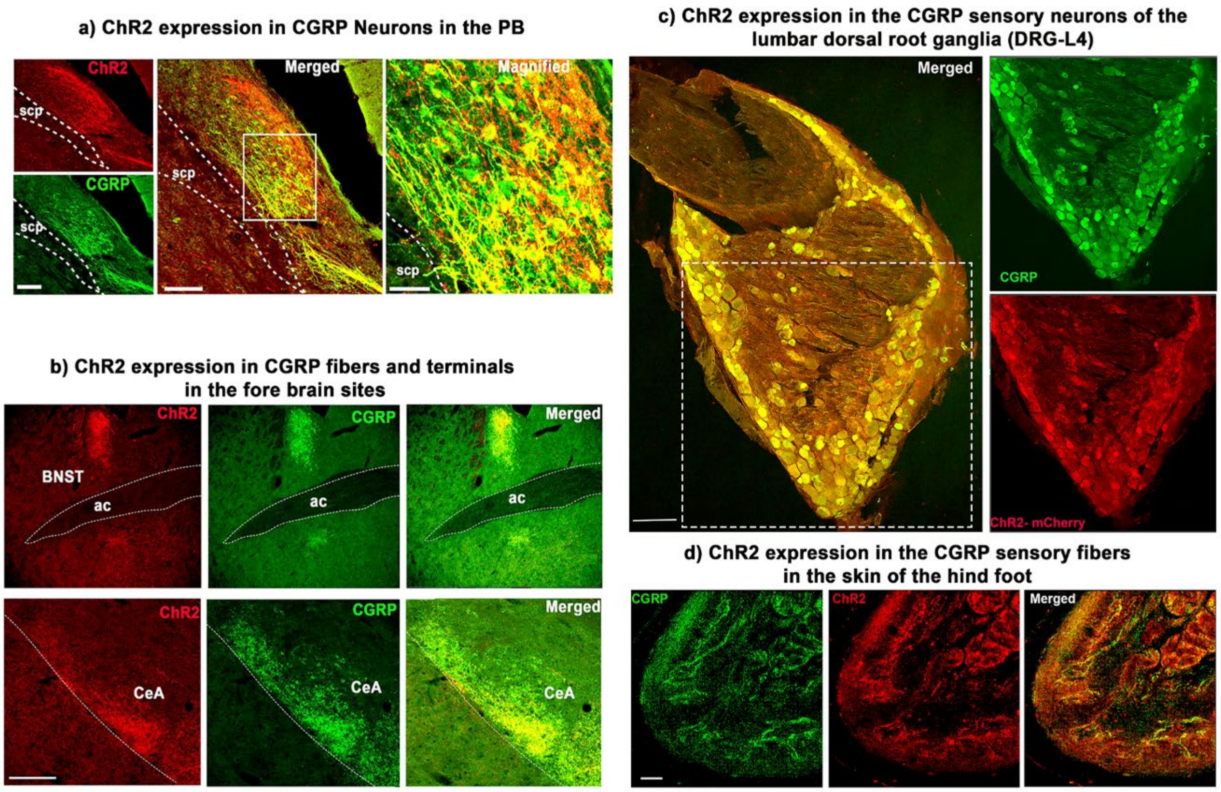

Figure S2

Supplementary Figure S2: *Channel Rhodopsin (ChR2) expression in CGRP-expressing neurons, terminals in the brain, and in sensory neurons of the dorsal root ganglia (DRG) and nerve endings in hind foot skin of CGRP-ChR2 mice:*

(**a, b**) Photomicrographs show brain sections from CGRP-ChR2 mice with immunolabeling of mCherry/ DsRed (red) for ChR2, and for CGRP (green) in PBel neurons and their terminals in BNST and CeA (Scale: 100μm in left panel of a, 60μm middle panel of a, and 30 μm right panel of a, 100μm in b).

c, d) ChR2/ DsRed (red) and CGRP (green) immunolabeling in the CGRP sensory neurons in the lumbar DRG (L4) and the nerve endings in the skin from the hind footpad (Scale: 100μm in c, 50μm in d), validating the presence of ChR2 in CGRP-ChR2 mice.

Abbreviations: ac, anterior commissure; BNST, bed nucleus of the stria terminalis; CeA, the central nucleus of the amygdala; scp, superior cerebellar peduncle.
